# Panorama of risky sexual behaviors in the Brazilian adult population – PNS 2019

**DOI:** 10.11606/s1518-8787.2022056004007

**Published:** 2022-06-20

**Authors:** Nayara Lopes Gomes, Claudia de Souza Lopes

**Affiliations:** I Universidade do Estado do Rio de Janeiro Instituto de Medicina Social Rio de Janeiro RJ Brasil Universidade do Estado do Rio de Janeiro. Instituto de Medicina Social. Rio de Janeiro, RJ, Brasil; II Instituto Brasileiro de Geografia e Estatística Diretoria de Pesquisas Coordenação de Trabalho e Rendimento Rio de Janeiro RJ Brasil Instituto Brasileiro de Geografia e Estatística. Diretoria de Pesquisas, Coordenação de Trabalho e Rendimento.Rio de Janeiro, RJ, Brasil

**Keywords:** Adult, Sexual Behavior, Health Risk Behaviors, Unsafe Sex, Socioeconomic Factors, Health Surveys

## Abstract

**OBJECTIVE:**

To describe the risky sexual behaviors of Brazilian adults according to socioeconomic, demographic, and regional characteristics.

**METHODS:**

Data from the 2019 National Health Survey, referring to the population aged 18 years or older, were analyzed. Risky sexual behaviors were considered: early sexual initiation, before the age of 15 years, and nonuse of condoms in the last sexual intercourse. Prevalence and respective confidence intervals were calculated for the subgroups of interest.

**RESULTS:**

Early sexual initiation among adult individuals was 24% among men and 11% among women, being higher among young people with lower levels of education and household income. The nonuse of condoms was higher among married/cohabiting partners, no schooling or with some elementary school, and among older people. The prevalence of nonuse of condoms among married/cohabiting partners was similar in both sexes (75.1% and 75.3%, among men and women). However, among non-cohabiting partners, gender disparity was relevant, as 34.1% of women did not use condoms in the last sexual intercourse, while among men this result was 21.3%.

**CONCLUSIONS:**

Higher prevalence of early sexual initiation for younger generations is noteworthy, especially among women. Concerning the nonuse of condoms, there are important gender disparities in the group of non-cohabiting partners, in addition to the high prevalence among older people, which should be considered in the formulation of public policies. The results of the present study are extremely relevant for understanding the adult population currently more vulnerable to sexually transmitted infections, after over five years without official statistics on this matter at the national level.

## INTRODUCTION

Risky sexual behaviors are related to sexual practices that can harm people’s health, especially sexual and reproductive, as they make them more vulnerable to sexually transmitted infections (STIs) and unintended pregnancy^1,2^.

Condom use in the last sexual intercourse and the age of sexual initiation are important indicators for monitoring populations at risk for human immunodeficiency virus (HIV) and other STIs. It is believed that information on condom use in the last sexual intercourse is easier and faster to collect, and with greater precision, when compared with that of consistent use, besides being considered a good proxy for condom use in general^3,4^. Conversely, the age of sexual initiation is usually investigated considering that early sexual initiation would increase the chances of problems such as abortion, STIs contagion, sexual abuse, and unintended pregnancy^5^.

For scientific-research purposes, sexual behaviors are investigated in different ways throughout the world in different target populations. Some research are dedicated to populations at higher risk, such as adolescents and sex workers, but the literature focused on the adult population is relatively scarce^6,7^.

The first academic investigations on risky sexual behaviors date back to 18^th^ century^8^. In Brazil, the first studies on the topic, with national scope for the adult population, date back to the 1990s, in view of the increase in the number of Aids cases at that time^9^. However, there are still few population-based surveys aimed at this population in the country.

The last study with national scope was conducted by the Brazilian Ministry of Health in 2013, focusing on people aged between 15 and 64 years^10^. Since then, more than five years have passed without nationwide information on the topic, until the *Pesquisa Nacional de Saúde* (PNS – National Health Survey) was held in 2019 and included, for the first time, a specific module on sexual activity.

Thus, the research is a unique opportunity to develop a more current and reliable panorama of sexual behaviors of the Brazilian adult population, at a time when recent studies point to the increase in STIs in recent years, in Brazil and other countries, such as the United States of America, in which an increase of 30% was observed between 2015 and 2019^11^.

According to data from the latest epidemiological bulletin on HIV/Aids, we can verify, for instance, a 75% increase in the Aids detection rate among men aged 20 to 24 years between 2009 and 2019^2^. Moreover, a significant trend of growth in syphilis, especially of the acquired type, is noteworthy, which recorded a 113% increase in the detection rate between 2015 and 2019; this may be related, among the possible factors, to the reduction in condom use^12,13^.

In this sense, efforts are extremely important to understand and outline the profile of people most susceptible to condom nonuse as well as to earlier sexual initiation.

The objective of this study was to describe the risky sexual behaviors of Brazilian adults in general and according to socioeconomic, demographic, and housing characteristics (macroregions and urban/rural regions), aiming to support targeted and more effective policies to prevent STIs.

## METHODS

This is a cross-sectional study, which used data from the second edition of the National Health Survey (PNS), conducted in 2019, by the *Instituto Brasileiro de Geografia e Estatística* (IBGE – Brazilian Institute of Geography and Statistics), in partnership with the Ministry of Health.

**Figure 1:**
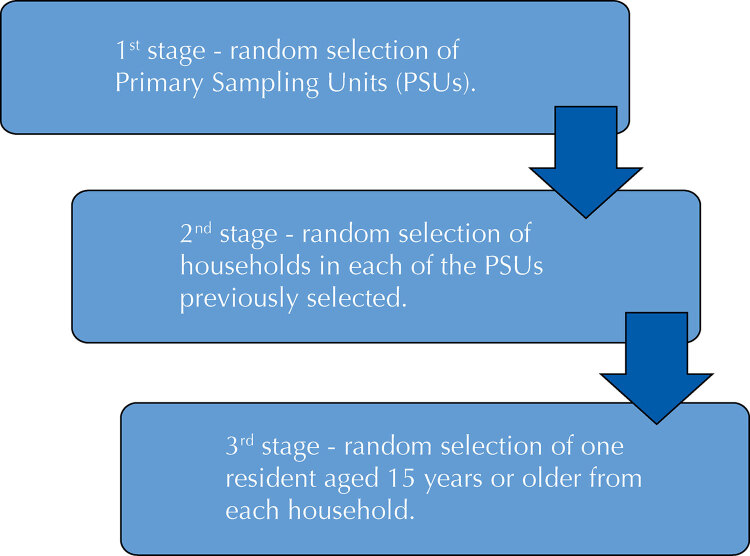
Flowchart of the sample selection process of the *Pesquisa Nacional de Saúde* (PNS – National Health Survey), 2019.

PNS is a nationwide household survey in which the sampling plan considered was clustered in three stages according to the flowchart presented in Figure 1^14^. The residents of the household responded to the research modules and, randomly, a single resident was selected to respond to specific modules as well, among which is the new module of sexual activity, object of the present study, answered only in case the selected resident was 18 years of age or older.

In this study, nonuse of condoms in the last sexual intercourse and early sexual initiation, assessed based on the age of sexual initiation, were considered as risky sexual behavior.

Adults aged 18 years or older were included in the analysis, totaling 88,531 interviewees^15^. To evaluate condom use, those who stated that they did not know or did not remember if they used condoms in the last sexual intercourse and those who refused to give this information were excluded. In the case of the evaluation of early sexual initiation, those who were not yet sexually active and who did not know or refused to answer the question were excluded.

Specifically for the analyses of early sexual initiation, according to socioeconomic characteristics, participants over 24 years of age were also excluded, aiming to reduce biases related to the difference between the current characteristics of the interviewees such as the situation of the household and those observed at the time of sexual initiation.

To evaluate early sexual initiation among different age cohorts, an analysis of the population aged 18 years or older was also performed, according to different age groups.

Condom use in the last sexual intercourse was investigated among people who claimed to have had relationships in the last 12 months, and who reported having used condoms with some frequency during this period, through the question: “In the past twelve months, in your last sexual intercourse, did you use a male or female condom?” Thus, the nonuse of condoms was obtained based on the people who answered “No” to this question and those who, for filter question purpose, did not respond to this question because they reported never having used condoms in sexual intercourses in the last 12 months.

Early sexual initiation was considered the situation in which the first sexual intercourse took place before 15 years of age, a criterion frequently used in the literature^16,17^. The age of sexual initiation was assessed by the question: “How old were you when you had sex for the first time?”

It is noteworthy that, in the PNS, vaginal and anal sex or oral sex with people of the same sex or of the opposite sex were considered sexual intercourse^18^.

The socioeconomic, demographic, and regional variables considered in the analyses were: i) skin color or ethnicity, as reported by the participants (self-reported), and only the results of those who self-reported being black, mixed-race, and white were tabled, considering that the estimates for Asian and Indigenous peoples present great inaccuracy; (ii) age groups divided between: 18 to 24 years, 25 to 29 years, 30 to 39 years, 40 to 49 years, 50 to 59 years, and 60 years or older; iii) level of education, obtained from the highest degree achieved by the interviewees, thus divided into: no schooling or some elementary school, elementary school or some high school, high school or some college, and college degree; iv) per capita household income (PCHI) ranges in minimum wages (MW): up to 1 MW, more than 1 to 3 MW, more than 3 to 5 MW, and more than 5 MW; v) whether or not the individual is employed (only considered for the analysis of nonuse of condoms); vi) macroregions of the country; and vii) situation of the household, whether urban or rural.

The prevalence and the respective 95% confidence intervals (IC95%) of the nonuse of condoms in the last sexual intercourse and early sexual initiation were estimated, considering the sample weight of the survey. All indicators were analyzed stratified by sex, according to socioeconomic and demographic variables. For the indicator of condom use, in addition to the mentioned strata, people who were married or cohabiting partners were analyzed separately from the others.

The comparison of the prevalence obtained between the different strata of the population and the evaluation of statistically significant differences were made based on the confidence intervals generated. Results whose confidence intervals were not overlapping were considered statistically different, considering a significance level of 5%.

Results of the statistical analyses were obtained from the SUDAAN and SAS Enterprise Guide version 8.1 softwares, considering the sample design of the survey.

The PNS was approved by the National Commission of Ethics in Research (Process no. 3.529.376 of August 23, 2019). The participants’ consent was obtained in two stages: first, at the beginning of the interview with residents of the household; and second, in the interview with the selected resident^14^.

## RESULTS

A total of 88,531 selected residents aged 18 years or older were interviewed at PNS 2019. Of these, 62,223 (70.3%) reported having had sex in the last 12 months, among which 769 (1.2%) refused to answer about condom use in these intercourses and 131 (0.2%) did not know or did not remember whether they used a condom or not, thus remaining a total of 61,323 people with information on condom use in the last sexual intercourse (Tables 1 and 2).

**Table 1:** Prevalence of nonuse of condoms in the last sexual intercourse and respective 95% confidence intervals of people aged 18 years or older, non-cohabiting, by sex, according to socioeconomic, demographic, and regional characteristics. *Pesquisa Nacional de Saúde* (PNS – National Health Survey), Brazil, 2019.

Socioeconomic, demographic, and regional characteristics	Non-cohabiting					
	Total		Men (n = 10,076)		Women (n = 9,006)	
Total	32.7	(31.5–34.0)	26.9	(25.2–28.5)	39.1	(37.3–41.0)
Age group						
18–24 years old	19.9	(17.5–22.6)	15.2	(12.5–18.3)	26.2	(22.0–30.9)
25–29 years old	27.5	(24.1–31.2)	23.0	(18.6–28.0)	32.7	(27.9–38.0)
30–39 years old	31.6	(29.3–33.9)	22.6	(19.6–26.0)	39.6	(36.5–42.7)
40–49 years old	40.5	(37.7–43.3)	32.8	(29.0–36.8)	45.8	(42.1–49.6)
50–59 years old	50.0	(46.7–53.3)	45.4	(40.7–50.1)	55.2	(50.8–59.5)
≥ 60 years old	59.7	(56.1–63.3)	55.7	(51.4–60.0)	67.6	(61.1–73.4)
Skin color or ethnicity						
White	34.6	(32.7–36.6)	28.5	(26.0–31.1)	40.9	(37.9–43.9)
Black	31.3	(28.0–34.8)	23.7	(19.4–28.6)	39.4	(34.2–44.7)
Mixed-race	31.5	(29.8–33.4)	26.6	(24.2–29.1)	37.4	(34.9–40.0)
Level of education						
No schooling or some elementary school	43.0	(40.6–45.4)	36.6	(33.5–39.7)	52.2	(48.4–56.0)
Elementary school or some high school	28.9	(25.9–32.1)	21.8	(18.3–25.7)	39.4	(34.7–44.2)
High school or some college	28.8	(26.9–30.9)	23.8	(21.2–26.5)	34.0	(31.1–37.1)
College degree	34.3	(31.6–37.1)	27.2	(23.5–31.3)	39.5	(35.9–43.2)
Per capita household income						
Up to 1 MW	32.0	(30.2–33.8)	24.5	(22.0–27.1)	38.6	(36.1–41.1)
More than 1 to 3 MW	32.7	(30.7–34.8)	27.6	(25.0–30.3)	39.7	(36.4–43.1)
More than 3 to 5 MW	33.2	(28.9–37.9)	29.9	(24.8–35.6)	37.6	(30.5–45.2)
More than 5 MW	37.4	(33.4–41.5)	33.5	(28.4–38.9)	43.0	(36.7–49.6)
Have an employment						
Yes	32.0	(30.5–33.5)	27.1	(25.2–29.0)	37.8	(35.6–40.1)
No	34.6	(32.3–36.9)	26.2	(23.3–29.4)	42.3	(38.9–45.7)
Household situation						
Urban	32.6	(31.2–34.0)	26.7	(24.9–28.6)	38.7	(36.7–40.7)
Rural	33.9	(31.1–36.9)	27.6	(24.6–30.9)	45.2	(40.1–50.4)
Macroregions						
North	23.7	(21.4–26.1)	19.0	(16.2–22.1)	29.4	(26.1–32.9)
Northeast	33.8	(31.9–35.7)	26.7	(24.5–28.9)	41.5	(38.5–44.5)
Southeast	33.1	(30.7–35.5)	28.0	(24.9–31.3)	38.6	(35.2–42.2)
South	33.8	(31.3–36.4)	26.8	(23.6–30.3)	41.2	(37.3–45.2)
Midwest	34.3	(30.9–37.9)	29.0	(24.6–33.9)	40.0	(35.8–44.4)

MW: minimum wage.

**Table 2:** Prevalence of nonuse of condoms in the last sexual intercourse and respective 95% confidence intervals of people aged 18 years or older, married or cohabitating, by sex, according to socioeconomic, demographic, and regional characteristics. *Pesquisa Nacional de Saúde* (PNS – National Health Survey), Brazil, 2019.

Socioeconomic, demographic, and regional characteristics	Married or cohabiting					
	Total		Men (n = 23,010)		Women (n = 19,231)	
Total	75.0	(74.3–75.7)	75.0	(74.0–75.9)	75.0	(74.0–76.1)
Age group						
18–24 years old	56.4	(53.3–59.4)	55.7	(50.7–60.7)	56.8	(53.2–60.4)
25–29 years old	62.7	(60.1–65.2)	62.1	(58.7–65.4)	63.2	(59.5–66.7)
30–39 years old	70.6	(69.2–72.1)	68.9	(66.9–70.9)	72.3	(70.3–74.3)
40–49 years old	75.4	(74.1–76.7)	74.7	(72.7–76.7)	76.1	(74.2–78.0)
50–59 years old	83.8	(82.4–85.1)	82.4	(80.5–84.1)	85.6	(83.5–87.5)
≥ 60 years old	89.8	(88.5–91.0)	88.5	(86.9–89.9)	92.4	(89.8–94.4)
Skin color or ethnicity						
White	77.0	(75.9–78.0)	77.4	(75.9–78.7)	76.6	(75.0–78.1)
Black	72.3	(70.1–74.4)	71.7	(68.6–74.6)	73.0	(69.8–76.0)
Mixed-race	73.8	(72.8–74.8)	73.5	(72.2–74.8)	74.1	(72.6–75.6)
Level of education						
No schooling or some elementary school	80.0	(78.9–81.1)	79.0	(77.6–80.4)	81.4	(79.6–83.1)
Elementary school or some high school	73.3	(71.4–75.2)	73.1	(70.6–75.5)	73.6	(70.7–76.3)
High school or some college	71.3	(70.1–72.5)	71.4	(69.6–73.1)	71.3	(69.5–72.9)
College degree	75.0	(73.5–76.5)	75.8	(73.3–78)	74.4	(72.2–76.5)
Per capita household income						
Up to 1 MW	73.2	(72.2–74.2)	72.6	(71.2–73.9)	73.8	(72.4–75.2)
More than 1 to 3 MW	76.5	(75.4–77.6)	76.8	(75.3–78.3)	76.1	(74.3–77.8)
More than 3 to 5 MW	76.8	(74.0–79.5)	78.3	(74.3–81.7)	75.0	(70.6–79.0)
More than 5 MW	79.5	(77.0–81.8)	78.8	(75.4–81.9)	80.3	(76.3–83.8)
Have an employment						
Yes	73.7	(72.8–74.5)	73.4	(72.3–74.5)	74.1	(72.8–75.4)
No	78.1	(76.9–79.3)	82.0	(80.0–83.8)	76.3	(74.7–77.8)
Household situation						
Urban	74.4	(73.6–75.2)	74.7	(73.6–75.8)	74.2	(72.9–75.3)
Rural	78.1	(76.8–79.3)	76.3	(74.6–78.0)	80.3	(78.6–81.8)
Macroregions						
North	66.1	(64.1–68.0)	65.0	(62.2–67.6)	67.3	(64.9–69.6)
Northeast	73.3	(72.1–74.4)	72.5	(70.9–74.0)	74.1	(72.6–75.6)
Southeast	76.5	(75.2–77.8)	76.6	(74.8–78.3)	76.4	(74.3–78.3)
South	77.1	(75.5–78.6)	77.8	(75.8–79.7)	76.2	(73.9–78.4)
Midwest	77.7	(76.1–79.3)	79.1	(76.9–81.2)	76.2	(73.9–78.4)

MW: minimum wage.

Of the total number of interviewees, 69,331 people reported the age of their sexual initiation, while 13,021 (14.7%) did not know how to give this information or did not remember it; 1,624 (1.8%) had never had sex in their lives; and 4,555 (5.1%) refused to answer the question. In the age group of 18 to 24 years, the total number of interviewees with information on sexual initiation was 5,955 people of both sexes (Table 3).

**Table 3:** Prevalence of early sexual initiation and respective 95% confidence intervals of people aged 18 to 24 years, by sex, according to socioeconomic, demographic, and regional characteristics. *Pesquisa Nacional de Saúde* (PNS – National Health Survey), Brazil, 2019.

Socioeconomic and demographic characteristics	Total	Men (n = 2,764)	Women (n = 3,191)
Total	24.2 (22.4–26.1)	28.5 (25.6–31.6)	19.9 (18.0–22.1)
Skin color or ethnicity			
White	19.5 (16.6–22.7)	23.5 (19.0–28.6)	15.7 (12.6–19.4)
Black	25.7 (21.1–30.9)	32.7 (25.2–41.1)	19.6 (14.1–26.7)
Mixed-race	27.5 (25.0–30.2)	31.6 (27.5–36.0)	23.3 (20.4–26.4)
Others	26.9 (13.2–47.1)	22.0 (6.4–53.8)	31.8 (16.3–52.7)
Level of education			
No schooling or some elementary school	46.7 (41.1–52.4)	46.4 (38.3–54.7)	47.0 (40.0–54.2)
Elementary school or some high school	32.3 (28.3–36.5)	36.6 (31–42.5)	27.2 (22.4–32.6)
High school or more	17.2 (15.1–19.5)	21.0 (17.6–24.9)	13.9 (11.7–16.5)
Per capita household income			
Up to 1 MW	27.9 (25.5–30.4)	32.9 (28.9–37.2)	23.3 (20.7–26.1)
More than 1 to 3 MW	20.2 (17.3–23.4)	24.3 (20–29.2)	15.9 (12.3–20.3)
More than 3 to 5 MW	11.5 (5.8–21.4)	15.4 (6.8–31.2)	6.1 (2.2–15.8)
More than 5 MW	8.0 (3.6–16.9)	12.3 (5.3–26.1)	0.6 (0.1–2.6)
Household situation			
Urban	24.2 (22.2–26.3)	28.9 (25.7–32.4)	19.6 (17.4–22.0)
Rural	24.1 (20.7–27.8)	26.0 (21.1–31.5)	22.0 (17.6–27.1)
Macroregions			
North	25.8 (22.6–29.3)	31.4 (26.2–37.2)	20.4 (16.7–24.8)
Northeast	28.1 (25.2–31.3)	32.7 (27.9–37.8)	23.6 (20.0–27.8)
Southeast	21.7 (18.4–25.5)	28.0 (22.6–34.1)	15.6 (12.3–19.4)
South	20.9 (17.3–25.0)	21.1 (15.9–27.6)	20.7 (15.7–26.7)
Midwest	27.5 (22.7–33.0)	26.6 (20.2–34.1)	28.4 (21.7–36.3)

MW: minimum wage.

Regarding the nonuse of condoms, the prevalence was separately estimated for non-cohabiting people and those who were married or cohabiting and by sex (Tables 1 and 2). The nonuse of condoms was considerably higher among married or cohabiting people, reaching 75% (95%CI: 74.3–75.7) in both sexes. As for non-cohabiting people, this percentage was 26.9% (95%CI: 25.2–28.5) and 39.1% (95%CI: 37.3–41.0), for men and women, respectively.

Regardless of marital status, nonuse of condoms was significantly higher among the older age groups and people with no schooling or with some elementary school. It is worth mentioning that, among women who are married or cohabiting, aged 60 years or older, we observed the highest prevalence of nonuse of condoms (92.4%; 95%CI: 89.8–94.4) among all analyzed categories. Moreover, the nonuse of condoms was significantly higher among married/cohabiting women in the rural region (80.3%; 95%CI: 78.6–81.8) when compared with those in the urban region (74.2%; 95%CI: 72.9–75.3).

Regarding the nonuse of condoms, when comparing the categories of each of the other socioeconomic, demographic, and regional variables not previously mentioned, we found statistically significant differences in the following cases: i) married/cohabiting, white men, with a higher percentage of nonuse of condoms when compared with black and mixed-race men; ii) non-cohabiting men and married/cohabiting men and women with PCHI greater than five MW, presenting higher prevalence than those with PCHI of up to 1 MW; iii) married/cohabiting men, unemployed, with a higher prevalence than those who are employed; iv) people from the Northern region presenting nonuse of condom lower than people from other regions for both sexes and marital status.

Differences in the nonuse of condoms between men and women, for the numerous characteristics analyzed, were relevant in the group of non-cohabiting people. The prevalence of nonuse of condoms was significantly different between sexes in all analyzed categories, except for those referring to people aged 25 to 29 years and those living in households whose PCHI was above three MW (Table 1).

We highlight, for example, the disparity observed among people aged between 30 and 39 years, an age group in which the prevalence of nonuse of condom among men was 22.6% (95%CI: 19.6–26.0), while among women it reached 39.6% (95%CI: 36.5–42.7). Among people with elementary school or some high school, the values reach 21.8% (95%CI: 18.3–25.7) and 39.4% (95%CI: 34.7–44.2), respectively.

**Figure 2:**
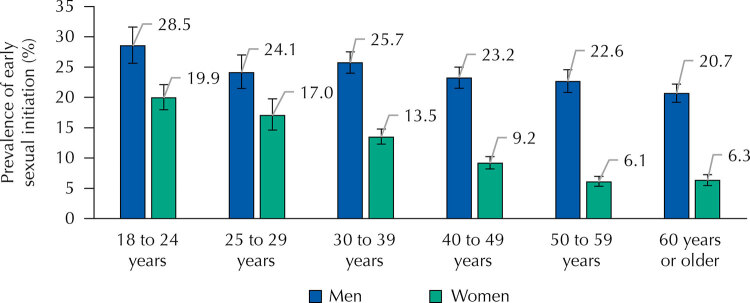
Prevalence of early sexual initiation and 95% confidence intervals of people aged 18 years or older, by sex, according to age groups. *Pesquisa Nacional de Saúde* (PNS – National Health Survey), Brazil, 2019.

As for sexual initiation, we found that men start sexual life earlier than women (Figure 2). In Brazil, approximately 24% of men aged 18 years or older started their sexual life early, while among women this prevalence reached 10.8%.

As we can observe in Figure 2, gender disparity has been decreasing with each generation. The difference in the prevalence of early sexual initiation between men and women was 16.5% and 14.4%, respectively, for people aged 50 to 59 years and 60 years or older. However, it decreases over the younger age groups, reaching 7.1% among people aged 25 to 29 years, and 8.6% among those aged 18 to 24 years.

Considering the population aged 18 to 24 years, the data also demonstrate that, for women, early sexual initiation is significantly higher also among mixed-race people, when compared with white people. Among mixed-race individuals, the prevalence reaches more than seven percentage points higher than that observed among white people (Table 3).

We also observed that, for both sexes, the lowest rates of early sexual initiation were among people with higher levels of education. While the prevalence was about 47% among those with no schooling or with some elementary school for both sexes, among people with high school or more, it was 21.0% and 13.9% for men and women, respectively.

We obtained similar results by analyzing early sexual initiation according to PCHI ranges, in which the lowest prevalence observed is in the group of women living in households with per capita income above 5 MW (0.6%; 95%CI: 0.1–2.6). In this group, the prevalence of early sexual initiation among men was 12.3% (95%CI: 5.3–26.1).

When evaluating the obtained results separately by macroregions of the country, we can highlight the Southeast region, where the prevalence of early sexual initiation among men was 28% (95%CI: 22.6–34.1), while among women it reached 15.6% (95%CI: 12.3–19.4).

## DISCUSSION

After more than five years without official statistics from government agencies on risky sexual behaviors of the adult population on a national scale, the inclusion of the new sexual activity module in PNS 2019 enabled to obtain a more current panorama on the subject, even allowing obtaining results for urban and rural regions as well as for the population of older adults.

To the best of our knowledge, the last research conducted on the topic was the *Pesquisa de Conhecimentos, Atitudes e Práticas da População Brasileira* (PCAP – Survey of Knowledge, Attitudes and Practices in the Brazilian Population), conducted by the Ministry of Health in 2013 (PCAP – 2013), focusing on the population aged between 15 and 64 years.

The results obtained in the present study indicate that the nonuse of condoms in the last sexual intercourse was significantly higher among people who were married or cohabiting with their partner (75%) and among older people, reaching, for example, 92.4% among married/cohabiting women aged 60 years or older, with the highest prevalence of all age groups and other characteristics evaluated.

The inclusion of older adults in the research is of great relevance, as an increase in the Aids detection rate among men aged 60 years or older has been observed in recent years, according to the latest epidemiological bulletin on the subject^2^.

The results presented in the PCAP – 2013 report, although with no breakdown by sex and marital status simultaneously, and being restricted to the population aged between 15 and 64 years, corroborate the fact that the nonuse of condoms is higher among married/cohabiting people, with lower levels of education, and more advanced age groups^10^.

In our study, for the group of non-cohabiting people, significant disparities were observed between sexes. These findings corroborate other studies on the adult population, in Brazil and other countries in North America, which point to a lower use of condoms among women^7,9,10,19,20^. Nevertheless, studies that evaluated adults simultaneously stratified by sex and marital status are rare.

The only study found in the literature is a national survey of people aged between 18 and 44 years in the USA, in which the nonuse of condoms among non-cohabiting women and men was 63.2% (95%CI: 61.1–65,2) and 49% (95%CI: 46.8–51.1) respectively. Although the confidence intervals were not overlapping between married/cohabiting men and women, the prevalence values were very similar, reaching 84.3% for men and 86.9% for women^21^.

Regarding early sexual initiation, our study greatly contributes by evaluating this outcome in the different generations of the population aged 18 years or older for both sexes.

When comparing the prevalence of early sexual initiation between men and women, we noticed a significant decrease between sexes for younger generations when compared with older generations. The decline in the age of menarche over generations, as well as changes in sexual norms, may explain this finding^22,23^.

The results of the PCAP – 2013, which used the same criterion of early sexual initiation before the age of 15 years, also indicate an earlier sexual initiation for younger generations. However, the analyzed age groups were more restricted and no analysis stratified by sex was performed^10^. Other population-based studies focused on adult people conducted in the USA and Thailand also corroborate this finding, in addition to the evidence of an increase in sexual partners throughout life for women from younger generations^23,24^.

We also observed the role of level of education and per capita household income in early sexual initiation. Individuals with lower levels of education and residents in households with lower PCHI presented higher prevalence of early sexual initiation. This result reinforces the importance of education in general and sex education in schools for the prevention of STIs among younger people, as already pointed out in other studies^17,24,25^.

As a strength of this study, we consider the fact that our data come from a population-based survey on a national scale, focusing on the adult population, which is less investigated in the topic of risky sexual behaviors. Furthermore, the outcome of condom use in the last sexual intercourse allows a better comparison with the results of other countries, as it is a measure widely used in surveys on the topic, in addition to being a good proxy for condom use over time^4,21^.

We also obtained results for the older adult population, unlike most other studies on this matter, because, overall, studies establish a maximum age limit during data collection. Finally, the analysis of condom use simultaneously stratified by sex and marital status is unprecedented. We identified no studies in Brazil for the adult population on a national scale with this design.

Among the limitations, we mention a possible, more intense memory bias for people of a higher age group, especially regarding the memory of the information on the age of sexual initiation. Moreover, the socioeconomic characteristics of the population aged between 18 and 24 years, informed at the time of collection, were considered proxy of those referring to the time of sexual initiation.

The aim of this study was to describe the risky sexual behaviors of Brazilian adults according to socioeconomic, demographic, and regional characteristics. The obtained results show relevant gender disparities. The high prevalence of nonuse of condoms in the population of older adults is noteworthy, as they are also exposed to STIs and must be considered in health promotion efforts. Lastly, the increased prevalence of early sexual initiation among women of younger generations is worrisome from the point of view of public health, and may imply an increase in unintended pregnancies and STIs.

Our results are extremely important to raise visibility to the strata of the adult population currently more vulnerable to STIs and to support future studies on the topic, in addition to indicating the need for public policies aimed at reducing gender disparities related to risky sexual behaviors.

## References

[1] Tu X, Lou C, Gao E, Li N, Zabin LS (2012). The relationship between sexual behavior and nonsexual risk behaviors among unmarried youth in three Asian cities. J Adolesc Health.

[2] Ministério da Saúde (BR), Secretaria de Vigilância em Saúde (2020). Bol Epidemiol HIV/Aids.

[3] Anderson JE, Wilson R, Doll L, Jones TS, Barker P (1999). Condom use and HIV risk behaviors among U.S. adults: data from a national survey. Fam Plann Perspect.

[4] Younge SN, Salazar LF, Crosby RF, DiClemente RJ, Wingood GM, Rose E (2008). Condom use at last sex as a proxy for other measures of condom use: is it good enough?. Adolescence.

[5] Yaya S, Bishwajit G (2018). Age at first sexual intercourse and multiple sexual partnerships among women in Nigeria: a cross-sectional analysis. Front Med.

[6] Dourado I, MacCarthy S, Reddy M, Calazans G, Gruskin S (2015). Revisitando o uso do preservativo no Brasil. Rev Bras Epidemiol.

[7] Fetner T, Dion M, Heath M, Andrejek N, Newell SL, Stick M (2020). Condom use in penile-vaginal intercourse among Canadian adults: results from the sex in Canada survey. PLoS One.

[8] Fenton KA, Johnson AM, McManus S, Erens B (2001). Measuring sexual behaviour: methodological challenges in survey research. Sex Transm Infect.

[9] Berquó E, Barbosa RM, Lima LP (2008). Uso do preservativo: tendências entre 1998 e 2005 na população brasileira. Rev Saude Publica.

[10] Ministério da Saúde (BR), Secretaria de Vigilância em Saúde, Departamento de DST, Aids e Hepatites Virais (2016). Pesquisa de conhecimentos, atitudes e práticas da população brasileira.

[11] Centers for Disease Control and Prevention (2021). NCHHSTP Newsroom. Press Release.

[12] Ministério da Saúde (BR), Secretaria de Vigilância em Saúde, Departamento de Doenças de Condições Crônicas e Infecções Sexualmente Transmissíveis (2020). Bol Epidemiol Sífilis.

[13] Santos MM, Lopes AKB, Roncalli AG, Lima KC (2020). Trends of syphilis in Brazil: a growth portrait of the treponemic epidemic. PLoS One.

[14] Stopa SR, Szwarcwald CL, Oliveira MM, Gouvea ECDP, Vieira MLFP, Freitas MPS (2020). Pesquisa Nacional de Saúde 2019: histórico, métodos e perspectivas. Epidemiol Serv Saude.

[15] Instituto Brasileiro de Geografia e Estatística, Diretoria de Pesquisas, Coordenação de Trabalho e Rendimento (2021). Pesquisa Nacional de Saúde 2019. Acidentes, violências, doenças transmissíveis, atividade sexual, características do trabalho e apoio social: Brasil.

[16] Magnusson BM, Crandall A, Evans K (2019). Early sexual debut and risky sex in young adults: the role of low self-control. BMC Public Health.

[17] Moraes L, Franca C, Silva B, Valença P, Menezes V, Colares V (2019). Iniciação sexual precoce e fatores associados: uma revisão de literatura. Psicol Saude Doenças.

[18] Instituto Brasileiro de Geografia e Estatística PNS - Pesquisa Nacional de Saúde, 2019. Conceitos e Métodos. Instrumentos de Coleta. PNS – Manual de Entrevista de Saúde.

[19] Herbenick D, Schick V, Reece M, Sanders SA, Smith N, Dodge B (2013). Characteristics of condom and lubricant use among a nationally representative probability sample of adults ages 18-59 in the United States. J Sex Med.

[20] Pascom ARP, Szwarcwald CL (2011). Sex inequalities in HIV-related practices in the Brazilian population aged 15 to 64 years old, 2008. Cad Saude Publica.

[21] Nasrullah M, Oraka E, Chavez PR, Johnson CH, DiNenno E (2017). Factors associated with condom use among sexually active US adults, National Survey of Family Growth, 2006-2010 and 2011-2013. J Sex Med.

[22] Ramraj B, Subramanian VM, Vijayakrishnan G (2021). Study on age of menarche between generations and the factors associated with it. Clin Epidemiol Glob Health.

[23] Techasrivichien T, Darawuttimaprakorn N, Punpuing S, Musumari PM, Lukhele BW, El-Saaidi C (2016). Changes in sexual behavior and attitudes across generations and gender among a population-based probability sample from an urbanizing province in Thailand. Arch Sex Behav.

[24] Liu G, Hariri S, Bradley H, Gottlieb SL, Leichliter JS, Markowitz LE (2015). Trends and patterns of sexual behaviors among adolescents and adults aged 14 to 59 years, United States. Sex Transm Dis.

[25] Shrestha R, Karki P, Copenhaver M (2016). Early sexual debut: a risk factor for STIs/HIV acquisition among a nationally representative sample of adults in Nepal. J Community Health.

