# Erratum

**DOI:** 10.11606/S01518-8787.2022056004007err

**Published:** 2022-11-18

In the article “Panorama of risky sexual behaviors in the Brazilian adult population – PNS 2019”, DOI https://doi.org/10.11606/s1518-8787.2022056004007, published on the Revista de Saúde Pública.2022;56:61, on pages 1,4,5,6,7 and 8 the RSP corrects tables 1 and 2, results section and estimates reported in the abstract and discussion.

**Where it reads:**

**Table:** Table 1 (p. 5):

Socioeconomic, demographic, and regional characteristics	Non-cohabiting					

	Total		Men (n = 10,076)		Women (n = 9,006)	
Total	32.7	(31.5–34.0)	26.9	(25.2–28.5)	39.1	(37.3–41.0)
Age group						
18–24 years old	19.9	(17.5–22.6)	15.2	(12.5–18.3)	26.2	(22.0–30.9)
25–29 years old	27.5	(24.1–31.2)	23.0	(18.6–28.0)	32.7	(27.9–38.0)
30–39 years old	31.6	(29.3–33.9)	22.6	(19.6–26.0)	39.6	(36.5–42.7)
40–49 years old	40.5	(37.7–43.3)	32.8	(29.0–36.8)	45.8	(42.1–49.6)
50–59 years old	50.0	(46.7–53.3)	45.4	(40.7–50.1)	55.2	(50.8–59.5)
≥ 60 years old	59.7	(56.1–63.3)	55.7	(51.4–60.0)	67.6	(61.1–73.4)
Skin color or ethnicity						
White	34.6	(32.7–36.6)	28.5	(26.0–31.1)	40.9	(37.9–43.9)
Black	31.3	(28.0–34.8)	23.7	(19.4–28.6)	39.4	(34.2–44.7)
Mixed-race	31.5	(29.8–33.4)	26.6	(24.2–29.1)	37.4	(34.9–40.0)
Level of education						
No schooling or some elementary school	43.0	(40.6–45.4)	36.6	(33.5–39.7)	52.2	(48.4–56.0)
Elementary school or some high school	28.9	(25.9–32.1)	21.8	(18.3–25.7)	39.4	(34.7–44.2)
High school or some college	28.8	(26.9–30.9)	23.8	(21.2–26.5)	34.0	(31.1–37.1)
College degree	34.3	(31.6–37.1)	27.2	(23.5–31.3)	39.5	(35.9–43.2)
Per capita household income						
Up to 1 MW	32.0	(30.2–33.8)	24.5	(22.0–27.1)	38.6	(36.1–41.1)
More than 1 to 3 MW	32.7	(30.7–34.8)	27.6	(25.0–30.3)	39.7	(36.4–43.1)
More than 3 to 5 MW	33.2	(28.9–37.9)	29.9	(24.8–35.6)	37.6	(30.5–45.2)
More than 5 MW	37.4	(33.4–41.5)	33.5	(28.4–38.9)	43.0	(36.7–49.6)
Have an employment						
Yes	32.0	(30.5–33.5)	27.1	(25.2–29.0)	37.8	(35.6–40.1)
No	34.6	(32.3–36.9)	26.2	(23.3–29.4)	42.3	(38.9–45.7)
Household situation						
Urban	32.6	(31.2–34.0)	26.7	(24.9–28.6)	38.7	(36.7–40.7)
Rural	33.9	(31.1–36.9)	27.6	(24.6–30.9)	45.2	(40.1–50.4)
Macroregions						
North	23.7	(21.4–26.1)	19.0	(16.2–22.1)	29.4	(26.1–32.9)
Northeast	33.8	(31.9–35.7)	26.7	(24.5–28.9)	41.5	(38.5–44.5)
Southeast	33.1	(30.7–35.5)	28.0	(24.9–31.3)	38.6	(35.2–42.2)
South	33.8	(31.3–36.4)	26.8	(23.6–30.3)	41.2	(37.3–45.2)
Midwest	34.3	(30.9–37.9)	29.0	(24.6–33.9)	40.0	(35.8–44.4)

**It should read:**

**Table:** Table 1 (p. 5):

Socioeconomic, demographic, and regional characteristics	Non–cohabiting					

	Total (n = 17,376)		Men (n = 9,162)		Women (n = 8,214)	
Total	27.3	(26.0–28.7)	21.3	(19.6–23.0)	34.1	(32.2–36.1)
Age group						
18–24 years old	19.8	(17.4–22.6)	15.2	(12.5–18.3)	26.1	(21.9–30.7)
25–29 years old	26.6	(23.2–30.4)	22.4	(18.0–27.4)	31.8	(26.8–37.2)
30–39 years old	27.6	(25.3–29.9)	18.9	(16.0–22.1)	35.5	(32.3–38.8)
40–49 years old	32.4	(29.8–35.1)	23.4	(20.1–27.1)	38.7	(35.0–42.6)
50–59 years old	39.9	(36.5–43.3)	34.1	(29.8–38.8)	46.0	(41.2–51.0)
≥ 60 years old	43.8	(39.8–47.9)	39.9	(35.4–44.5)	52.0	(44.2–59.8)
Skin color or ethnicity						
White	28.9	(26.8–31.0)	22.7	(20.2–25.4)	35.3	(32.2–38.5)
Black	28.0	(24.7–31.5)	20.1	(15.9–25.1)	36.1	(30.9–41.5)
Mixed-race	25.9	(24.1–27.8)	20.6	(18.4–23.1)	32.2	(29.7–34.8)
Level of education						
No schooling or some elementary school	33.7	(31.2–36.3)	26.8	(23.8–30.0)	43.8	(39.8–48.0)
Elementary school or some high school	24.2	(21.2–27.4)	17.9	(14.5–21.8)	34.0	(29.6–38.8)
High school or some college	24.9	(22.9–27.0)	20.0	(17.4–22.9)	30.0	(27.0–33.1)
College degree	29.9	(27.2–32.8)	21.4	(18.0–25.2)	36.2	(32.4–40.1)
Per capita household income						
Up to 1 MW	27.5	(25.7–29.4)	18.9	(16.4–21.6)	34.9	(32.4–37.6)
More than 1 to 3 MW	26.5	(24.4–28.7)	22.2	(19.6–25.0)	32.7	(29.3–36.3)
More than 3 to 5 MW	27.8	(23.4–32.6)	24.3	(19.2–30.2)	32.5	(25.3–40.6)
More than 5 MW	30.2	(26.4–34.3)	26.7	(22.0–32.1)	35.4	(28.9–42.6)
Have an employment						
Yes	26.9	(25.4–28.6)	21.6	(19.8–23.6)	33.2	(30.8–35.6)
No	28.4	(26.2–30.8)	20.2	(17.4–23.3)	36.2	(32.8–39.8)
Household situation						
Urban	27.4	(26.0–28.8)	21.3	(19.5–23.2)	33.8	(31.7–35.8)
Rural	27.1	(24.3–30.2)	21.2	(18.3–24.5)	38.4	(33.0–44.0)
Macroregions						
North	20.5	(18.2–23.0)	16.1	(13.4–19.2)	25.9	(22.6–29.5)
Northeast	29.0	(27.1–31.1)	21.0	(18.8–23.3)	37.7	(34.7–40.9)
Southeast	28.1	(25.7–30.7)	22.9	(19.8–26.3)	33.9	(30.4–37.7)
South	25.3	(22.8–28.0)	19.2	(16.0–22.8)	32.2	(28.3–36.4)
Midwest	27.7	(24.3–31.4)	22.1	(17.9–27.0)	33.9	(29.7–38.2)

**Where it reads:**

**Table:** Table 2 (p. 6):

Socioeconomic, demographic, and regional characteristics	Married or cohabiting					

	Total		Men (n=23,010)		Women (n=19,231)	
Total	75.0	(74.3–75.7)	75.0	(74.0–75.9)	75.0	(74.0–76.1)
Age group						
18–24 years old	56.4	(53.3–59.4)	55.7	(50.7–60.7)	56.8	(53.2–60.4)
25–29 years old	62.7	(60.1–65.2)	62.1	(58.7–65.4)	63.2	(59.5–66.7)
30–39 years old	70.6	(69.2–72.1)	68.9	(66.9–70.9)	72.3	(70.3–74.3)
40–49 years old	75.4	(74.1–76.7)	74.7	(72.7–76.7)	76.1	(74.2–78.0)
50–59 years old	83.8	(82.4–85.1)	82.4	(80.5–84.1)	85.6	(83.5–87.5)
≥ 60 years old	89.8	(88.5–91.0)	88.5	(86.9–89.9)	92.4	(89.8–94.4)
Skin color or ethnicity						
White	77.0	(75.9–78.0)	77.4	(75.9–78.7)	76.6	(75.0–78.1)
Black	72.3	(70.1–74.4)	71.7	(68.6–74.6)	73.0	(69.8–76.0)
Mixed-race	73.8	(72.8–74.8)	73.5	(72.2–74.8)	74.1	(72.6–75.6)
Level of education						
No schooling or some elementary school	80.0	(78.9–81.1)	79.0	(77.6–80.4)	81.4	(79.6–83.1)
Elementary school or some high school	73.3	(71.4–75.2)	73.1	(70.6–75.5)	73.6	(70.7–76.3)
High school or some college	71.3	(70.1–72.5)	71.4	(69.6–73.1)	71.3	(69.5–72.9)
College degree	75.0	(73.5–76.5)	75.8	(73.3–78)	74.4	(72.2–76.5)
Per capita household income						
Up to 1 MW	73.2	(72.2–74.2)	72.6	(71.2–73.9)	73.8	(72.4–75.2)
More than 1 to 3 MW	76.5	(75.4–77.6)	76.8	(75.3–78.3)	76.1	(74.3–77.8)
More than 3 to 5 MW	76.8	(74.0–79.5)	78.3	(74.3–81.7)	75.0	(70.6–79.0)
More than 5 MW	79.5	(77.0–81.8)	78.8	(75.4–81.9)	80.3	(76.3–83.8)
Have an employment						
Yes	73.7	(72.8–74.5)	73.4	(72.3–74.5)	74.1	(72.8–75.4)
No	78.1	(76.9–79.3)	82.0	(80.0–83.8)	76.3	(74.7–77.8)
Household situation						
Urban	74.4	(73.6–75.2)	74.7	(73.6–75.8)	74.2	(72.9–75.3)
Rural	78.1	(76.8–79.3)	76.3	(74.6–78.0)	80.3	(78.6–81.8)
Macroregions						
North	66.1	(64.1–68.0)	65.0	(62.2–67.6)	67.3	(64.9–69.6)
Northeast	73.3	(72.1–74.4)	72.5	(70.9–74.0)	74.1	(72.6–75.6)
Southeast	76.5	(75.2–77.8)	76.6	(74.8–78.3)	76.4	(74.3–78.3)
South	77.1	(75.5–78.6)	77.8	(75.8–79.7)	76.2	(73.9–78.4)
Midwest	77.7	(76.1–79.3)	79.1	(76.9–81.2)	76.2	(73.9–78.4)

**It should read:**

**Table:** Table 2 (p. 6):

Socioeconomic, demographic, and regional characteristics	Married or cohabiting					

	Total (n = 43,947)		Men (n = 23,924)		Women (n = 20,023)	
Total	75.2	(74.5–75.9)	75.1	(74.2–76.1)	75.3	(74.2–76.3)
Age group						
18–24 years old	56.4	(53.3–59.4)	55.7	(50.7–60.6)	56.8	(53.1–60.4)
25–29 years old	62.7	(60.2–65.2)	62.3	(58.9–65.5)	63.1	(59.5–66.6)
30–39 years old	70.7	(69.2–72.1)	68.9	(66.8–70.8)	72.5	(70.5–74.4)
40–49 years old	75.5	(74.2–76.8)	74.8	(72.8–76.7)	76.1	(74.2–78.0)
50–59 years old	83.7	(82.0–85.0)	82.2	(80.1–84.1)	85.7	(83.7–87.5)
≥ 60 years old	89.8	(88.6–90.9)	88.4	(86.9–89.8)	92.5	(90.1–94.4)
Skin color or ethnicity						
White	77.2	(76.2–78.2)	77.5	(76.1–78.8)	76.9	(75.3–78.4)
Black	72.2	(69.8–74.4)	71.2	(67.9–74.3)	73.3	(70.2–76.2)
Mixed-race	74.1	(73.1–75.1)	73.9	(72.6–75.2)	74.3	(72.8–75.7)
Level of education						
No schooling or some elementary school	80.3	(79.2–81.4)	79.3	(77.9–80.6)	81.7	(79.9–83.3)
Elementary school or some high school	73.1	(71.2–74.9)	72.6	(70.1–75.0)	73.6	(70.8–76.3)
High school or some college	71.6	(70.4–72.8)	71.5	(69.8–73.2)	71.7	(70.0–73.3)
College degree	75.2	(73.7–76.7)	76.2	(73.8–78.4)	74.4	(72.2–76.5)
Per capita household income						
Up to 1 MW	73.4	(72.4–74.4)	72.9	(71.5–74.1)	74.0	(72.6–75.3)
More than 1 to 3 MW	76.6	(75.4–77.7)	76.8	(75.1–78.4)	76.4	(74.6–78.1)
More than 3 to 5 MW	77.0	(74.2–79.6)	78.5	(74.7–81.9)	75.0	(70.8–78.9)
More than 5 MW	80.1	(77.7–82.3)	79.4	(76.0–82.4)	81.0	(77.2–84.2)
Have an employment						
Yes	73.9	(73.0–74.7)	73.5	(72.4–74.6)	74.3	(73.0–75.6)
No	78.5	(77.2–79.6)	82.3	(80.4–84.0)	76.6	(75.1–78.1)
Household situation						
Urban	74.6	(73.9–75.4)	74.8	(73.7–75.9)	74.4	(73.3–75.6)
Rural	78.4	(77.1–79.6)	76.7	(74.9–78.4)	80.4	(78.8–82.0)
Macroregions						
North	66.3	(64.4–68.2)	65.2	(62.5–67.8)	67.6	(65.3–69.9)
Northeast	73.6	(72.4–74.7)	72.8	(71.3–74.3)	74.4	(72.9–75.9)
Southeast	76.5	(75.3–77.7)	76.6	(74.7–78.3)	76.5	(74.5–78.4)
South	77.3	(75.7–78.8)	78.0	(76.0–79.9)	76.6	(74.3–78.7)
Midwest	78.2	(76.6–79.7)	79.5	(77.4–81.5)	76.7	(74.4–78.7)

**Where it reads:**

Abstract (p. 1):

“The prevalence of nonuse of condoms among married/cohabiting partners was the same in both sexes (75%).”

“39.1%”; “26.9%.”

**It should read:**

Abstract (p. 1):

“The prevalence of nonuse of condoms among married/cohabiting partners was similar in both sexes (75.1% and 75.3%, among men and women)”

“34.1%”; “21.3%.”

**Where it reads:**

Results section:

(p. 4, last paragraph)

“75% (95%CI: 74.3–75.7)”;“26.9% (95%CI: 25.2–28.5)”; “39.1% (95%CI: 37.3–41.0)”

**It should read:**

Results section:

(p. 4, last paragraph)

“75.2% (95%CI: 74.5–75.9)”;“21.3% (95%CI: 19.6–23.0)”; “34.1% (95%CI: 32.2–36.1)”

**Where it reads:**

(p. 5, 1^st^ paragraph)

“Regardless of marital status, nonuse of condoms was significantly higher among the older age groups and people with no schooling or with some elementary school.”

“(92.4%; 95%CI: 89.8–94.4)”; “(80.3%; 95%CI: 78.6–81.8)”; “(74.2%; 95%CI: 72.9–75.3)”

**It should read:**

(p. 5, 1^st^ paragraph)

“Regardless of marital status, nonuse of condoms was significantly higher among the older age groups.”

“(92.5%; 95%CI: 90.1–94.4)”; “(80.4%; 95%CI: 78.8–82.0)”; “(74.4%; 95%CI: 73.3–75.6).”

**Where it reads:**

(p. 7, 1^st^ paragraph)

“iv) people from the Northern region presenting nonuse of condom lower than people from other regions for both sexes and marital status.”

**It should read:**

(p. 7, 1^st^ paragraph)

“iv) people from the Northern region married or cohabiting presenting nonuse of condom lower than people from other regions for both sexes.”

**Where it reads:**

(p. 7, 2^nd^ paragraph)

“except for those referring to people aged 25 to 29 years and those living in households whose PCHI was above three MW (Table 1).”

**It should read:**

(p. 7, 2^nd^ paragraph)

“except for those referring to people aged 25 to 29 years, aged 60 years or older and those living in households whose PCHI was above three MW (Table 1).”

**Where it reads:**

(p. 7, 3^rd^ paragraph)

“We highlight, for example, the disparity observed among people aged between 30 and 39 years, an age group in which the prevalence of nonuse of condom among men was 22.6% (95%CI: 19.6–26.0), while among women it reached 39.6% (95%CI: 36.5–42.7). Among people with elementary school or some high school, the values reach 21.8% (95%CI: 18.3–25.7) and 39.4% (95%CI: 34.7–44.2), respectively.”

**It should read:**

(p. 7, 3^rd^ paragraph)

“We highlight, for example, the disparity observed among people living in rural areas, in which the prevalence of nonuse of condom among men was 21.2% (95%CI: 18.3–24.5), while among women it reached 38.4% (95%CI: 33.0–44.0). Among people with no schooling or some elementary school, the values reach 26.8% (95%CI: 23.8–30.0) and 43.8% (95%CI: 39.8–48.0), respectively.”

**Where it reads:**

Discussion section:

(p. 8, last paragraph)

“(75%)”; “92.4%”

**It should read:**

Discussion section:

(p. 8, last paragraph)

“(75.2%)”; “92.5%”

