# Supplementary material for: Panorama of risky sexual behaviors in the Brazilian adult population – PNS 2019
Source: Rev Saude Publica. 2022 Jun 20;56:61. doi: 10.11606/s1518-8787.2022056004007 (PMC9239426; doi:10.11606/s1518-8787.2022056004007)
Supplement: Translation

# Translation: Panorama dos comportamentos sexuais de risco na população adulta brasileira - PNS 2019

**Authors:** Nayara Lopes Gomes, Claudia de Souza Lopes

**Affiliations:** I Brasil Universidade do Estado do Rio de Janeiro. Instituto de Medicina Social. Rio de Janeiro, RJ, Brasil; II Brasil Instituto Brasileiro de Geografia e Estatística. Diretoria de Pesquisas, Coordenação de Trabalho e Rendimento.Rio de Janeiro, RJ, Brasil

**Keywords:** Adulto, Comportamento Sexual, Comportamentos de Risco à Saúde, Sexo sem Proteção, Fatores Socioeconômicos, Inquéritos Epidemiológicos

## Abstract

**OBJETIVO:**

Descrever os comportamentos sexuais de risco dos adultos brasileiros segundo características socioeconômicas, demográficas e regionais.

**MÉTODOS:**

Foram analisados dados da Pesquisa Nacional de Saúde 2019, referente à população de 18 anos de idade ou mais. Considerou-se como comportamentos sexuais de risco: a iniciação sexual precoce, antes dos 15 anos, e o não uso de preservativo na última relação sexual. Foram calculadas as prevalências e respectivos intervalos de confiança para os subgrupos de interesse.

**RESULTADOS:**

A iniciação sexual precoce entre indivíduos adultos foi de 24% entre os homens e 11% entre as mulheres, sendo mais alta entre jovens com menores níveis de instrução e rendimento domiciliar. O não uso de preservativo se mostrou maior entre os casados/coabitantes, sem instrução ou com nível fundamental incompleto, e entre os mais velhos. A prevalência do não uso de preservativo entre casados/coabitantes foi similar em ambos os sexos (75,1% e 75,3%, entre homens e mulheres). No entanto, entre os não coabitantes, a disparidade entre os sexos se mostrou relevante uma vez que 34,1% das mulheres não usaram preservativo na última relação sexual, enquanto entre os homens esse resultado foi de 21,3%.

**CONCLUSÃO:**

Nota-se, especialmente entre as mulheres, maiores prevalências de iniciação sexual precoce para as gerações mais novas. No que se refere ao não uso de preservativo, há disparidades de sexo importantes no grupo dos não coabitantes, além da alta prevalência entre os mais velhos, que devem ser consideradas na elaboração das políticas públicas. Os resultados do presente estudo são extremamente relevantes para compreensão da população adulta atualmente mais vulnerável às infecções sexualmente transmissíveis, após mais de cinco anos sem estatísticas oficiais a respeito em âmbito nacional.

## INTRODUÇÃO

Os comportamentos sexuais de risco estão relacionados a práticas sexuais que podem causar prejuízos à saúde das pessoas, especialmente sexual e reprodutiva, pois as tornam mais vulneráveis a infecções sexualmente transmissíveis (IST) e a gravidez indesejada^1,2^.

O uso de preservativo na última relação sexual e a idade de iniciação sexual, são indicadores importantes para o monitoramento de populações em risco para HIV e outras IST. Acredita-se que a informação do uso do preservativo na última relação sexual seja mais fácil e rápida de ser coletada, e com maior precisão, quando comparada a do uso consistente, além de ser considerada uma boa *proxy* para o uso de preservativos de uma maneira geral^3,4^. Já a idade de iniciação sexual costuma ser investigada tendo em vista que uma iniciação sexual precoce aumentaria as chances de problemas como aborto, contágio por IST, abuso sexual e gravidez não planejada^5^.

Para fins de estudos científicos, os comportamentos sexuais são investigados de diferentes formas pelo mundo em populações alvo distintas. Há uma concentração de pesquisas dedicadas às populações de maior risco como, por exemplo, adolescentes e profissionais do sexo, porém, a literatura focalizada na população adulta é relativamente escassa^6,7^.

As primeiras investigações acadêmicas sobre comportamentos sexuais de risco surgiram no século XVIII^8^. No Brasil, os primeiros estudos sobre o tema, com abrangência nacional para a população adulta, datam dos anos 1990, tendo em vista o aumento do número de casos de aids naquele momento^9^. No entanto, ainda são poucos os inquéritos de base populacional voltados para essa população no país.

O último estudo, com abrangência nacional, foi realizado pelo Ministério da Saúde no ano de 2013, com foco nas pessoas com idade entre 15 e 64 anos^10^. Desde então, passaram-se mais de cinco anos sem informações em âmbito nacional sobre o tema, até a Pesquisa Nacional de Saúde (PNS) ser realizada no ano 2019 incluindo, pela primeira vez, um módulo específico sobre atividade sexual.

Dessa forma, a pesquisa se configura como uma oportunidade única para a construção de um panorama mais atual e fidedigno dos comportamentos sexuais da população adulta brasileira, em um momento em que estudos recentes apontam para o aumento das IST nos últimos anos, no Brasil e em outros países, como nos EUA, em que foi constatado um crescimento de 30% entre 2015 e 2019^11^.

De acordo com os dados do último boletim epidemiológico de HIV/Aids, verifica-se, por exemplo, um aumento de 75% na taxa de detecção de aids entre homens de 20 a 24 anos, entre 2009 e 2019^2^. Há ainda uma tendência expressiva de crescimento da sífilis, especialmente da adquirida, a qual registrou um aumento de 113% na taxa de detecção entre 2015 e 2019, o que pode estar relacionado, dentre os possíveis fatores, à redução no uso de preservativo^12,13^.

Nesse sentido, são de extrema importância esforços para compreender e delinear o perfil das pessoas mais susceptíveis ao não uso do preservativo, bem como à iniciação sexual mais precoce.

O objetivo do presente estudo foi descrever os comportamentos sexuais de risco dos adultos brasileiros de forma geral e segundo características socioeconômicas, demográficas e região de moradia (macrorregiões e urbana/rural), visando subsidiar políticas direcionadas e mais eficazes de prevenção às IST.

## MÉTODOS

Trata-se de um estudo transversal, que utilizou dados da segunda edição da Pesquisa Nacional de Saúde (PNS), realizada em 2019, pelo Instituto Brasileiro de Geografia e Estatística (IBGE), em parceria com o Ministério da Saúde.

A PNS é uma pesquisa domiciliar de abrangência nacional em que o plano amostral utilizado foi por conglomerados em três estágios conforme fluxograma apresentado na Figura 1^14^. Os moradores do domicílio respondiam aos módulos da pesquisa e, de forma aleatória, um único morador era selecionado para responder também a módulos específicos, dentre os quais, encontra-se o novo módulo de atividade sexual, objeto de estudo desse artigo, respondido apenas no caso de o morador selecionado ter 18 anos de idade ou mais.

**Figura 1:** Fluxograma do processo de seleção amostral da Pesquisa Nacional de Saúde, 2019.

No presente estudo, foram considerados como comportamento sexual de risco o não uso de preservativo na última relação sexual e a iniciação sexual precoce, avaliada a partir da idade de iniciação sexual.

Foram incluídos na análise os adultos de 18 anos de idade ou mais, totalizando 88.531 entrevistados^15^. Para avaliação do uso do preservativo, foram excluídos aqueles que afirmaram não saber ou não se lembrar se usaram o preservativo na última relação sexual e os que se recusaram a dar essa informação. No caso da avaliação da iniciação sexual precoce, foram excluídos os que ainda não haviam iniciado a vida sexual e que não sabiam ou se recusaram a responder à pergunta.

Especificamente para as análises da iniciação sexual precoce, segundo características socioeconômicas, foram excluídos ainda os participantes com idade acima de 24 anos, visando diminuir os vieses relacionados à diferença entre as características atuais dos entrevistados, como, por exemplo, a situação do domicílio e aquelas observadas no momento da iniciação sexual.

Visando avaliar a iniciação sexual precoce entre as diferentes coortes de idade, foi realizada ainda uma análise da população de 18 anos de idade ou mais, segundo as diferentes faixas etárias.

O uso do preservativo na última relação sexual foi investigado entre as pessoas que afirmaram terem tido relações nos últimos 12 meses, e que informaram terem usado preservativo com alguma frequência nesse período, por meio da pergunta: “Nos últimos doze meses, na última relação sexual que teve, usou camisinha masculina ou feminina?” Sendo assim, o não uso do preservativo foi obtido com base nas pessoas que responderam “Não” a essa pergunta e naquelas que, pelo fluxo da pesquisa, não respondiam a esse quesito por informarem nunca terem usado preservativo nas relações sexuais tidas dos últimos 12 meses.

Foi considerada iniciação sexual precoce a situação em que a primeira relação sexual ocorreu antes dos 15 anos de idade, critério frequentemente utilizado na literatura^16,17^. A idade de iniciação sexual foi avaliada por meio da pergunta: “Que idade tinha quando teve relações sexuais pela primeira vez?”

Vale ressaltar que, na PNS, foi considerada relação sexual o sexo com penetração vaginal, anal ou sexo oral com pessoas do mesmo sexo ou do sexo oposto^18^.

As variáveis socioeconômicas, demográficas e regionais consideradas nas análises foram: i) cor ou raça, captada segundo a autodeclaração, sendo explicitados nas tabelas apenas os resultados dos pretos, pardos e brancos, tendo em vista que as estimativas para amarelos e indígenas apresentam grande imprecisão; ii) faixas etárias divididas entre: 18 a 24 anos, 25 a 29 anos, 30 a 39 anos, 40 a 49 anos, 50 a 59 anos e 60 anos ou mais; iii) nível de instrução, obtido a partir do maior grau alcançado pelos entrevistados, sendo dividido em: sem instrução ou fundamental incompleto, fundamental completo ou médio incompleto, médio completo ou superior incompleto e superior completo; iv) faixas de rendimento domiciliar per capita (RDPC) em salários mínimos (SM): até 1 SM, mais de 1 a 3 SM, mais de 3 a 5 SM e mais de 5 SM; v) se possui trabalho ou não (considerada apenas para a análise do não uso de preservativo) vi) grandes regiões do país; e vii) situação do domicílio, sendo urbano ou rural.

Foram calculadas as prevalências e os respectivos intervalos de confiança de 95% (IC95%) do não uso de preservativo na última relação sexual e da iniciação sexual precoce, considerando o peso amostral da pesquisa. Todos os indicadores foram analisados estratificados por sexo, segundo as variáveis socioeconômicas e demográficas. Para o indicador do uso de preservativo, foram analisadas ainda as pessoas casadas ou em coabitação separadamente das demais.

A comparação das prevalências obtidas entre os diferentes estratos da população e a avaliação de diferenças estatisticamente significativas foram realizadas com base nos intervalos de confiança gerados. Os resultados cujos intervalos de confiança não se encontravam sobrepostos foram considerados estatisticamente diferentes, considerando um nível de significância de 5%.

Os resultados das análises estatísticas foram obtidos por meio dos softwares SUDAAN e SAS Enterprise Guide versão 8.1, de forma a considerar o desenho amostral da pesquisa.

A PNS foi aprovada na Comissão Nacional de Ética em Pesquisa (Processo: 3.529.376, de 23 de agosto de 2019). O consentimento dos participantes foi obtido em duas etapas, sendo o primeiro no início da entrevista das pessoas do domicílio e o segundo na entrevista do morador selecionado^14^.

## RESULTADOS

Foram entrevistados na PNS 2019 um total de 88.531 moradores selecionados de 18 anos de idade ou mais. Desses, 62.223 (70,3%) informaram terem tido relações sexuais nos últimos 12 meses, dentre os quais 769 (1,2%) se recusaram a responder sobre o uso de preservativo nessas relações e 131 (0,2%) não souberam dizer ou não lembravam sobre o uso do preservativo, restando assim um total de 61.323 pessoas com a informação do uso de preservativo na última relação sexual (Tabelas 1 e 2).

**Tabela 1:** Prevalência do não uso de preservativo na última relação sexual e respectivos intervalos de confiança de 95% das pessoas de 18 anos ou mais não coabitantes, por sexo, segundo características socioeconômicas, demográficas e regionais. Pesquisa Nacional de Saúde, Brasil, 2019.

Características socioeconômicas, demográficas e regionais	Não coabitantes					
	Total		Homens (n = 10.076)		Mulheres (n = 9.006)	
Total	32,7	(31,5–34,0)	26,9	(25,2–28,5)	39,1	(37,3–41,0)
Faixa etária						
18–24 anos	19,9	(17,5–22,6)	15,2	(12,5–18,3)	26,2	(22,0–30,9)
25–29 anos	27,5	(24,1–31,2)	23,0	(18,6–28,0)	32,7	(27,9–38,0)
30–39 anos	31,6	(29,3–33,9)	22,6	(19,6–26,0)	39,6	(36,5–42,7)
40–49 anos	40,5	(37,7–43,3)	32,8	(29,0–36,8)	45,8	(42,1–49,6)
50–59 anos	50,0	(46,7–53,3)	45,4	(40,7–50,1)	55,2	(50,8–59,5)
≥ 60 anos	59,7	(56,1–63,3)	55,7	(51,4–60,0)	67,6	(61,1–73,4)
Cor ou raça						
Branca	34,6	(32,7–36,6)	28,5	(26,0–31,1)	40,9	(37,9–43,9)
Preta	31,3	(28,0–34,8)	23,7	(19,4–28,6)	39,4	(34,2–44,7)
Parda	31,5	(29,8–33,4)	26,6	(24,2–29,1)	37,4	(34,9–40,0)
Nível de instrução						
Sem instrução ou fundamental incompleto	43,0	(40,6–45,4)	36,6	(33,5–39,7)	52,2	(48,4–56,0)
Fundamental completo ou médio incompleto	28,9	(25,9–32,1)	21,8	(18,3–25,7)	39,4	(34,7–44,2)
Médio completo ou superior incompleto	28,8	(26,9–30,9)	23,8	(21,2–26,5)	34,0	(31,1–37,1)
Superior completo	34,3	(31,6–37,1)	27,2	(23,5–31,3)	39,5	(35,9–43,2)
Rendimento domiciliar per capita						
Até 1 SM	32,0	(30,2–33,8)	24,5	(22,0–27,1)	38,6	(36,1–41,1)
Mais de 1 a 3 SM	32,7	(30,7–34,8)	27,6	(25,0–30,3)	39,7	(36,4–43,1)
Mais de 3 a 5 SM	33,2	(28,9–37,9)	29,9	(24,8–35,6)	37,6	(30,5–45,2)
Mais de 5 SM	37,4	(33,4–41,5)	33,5	(28,4–38,9)	43,0	(36,7–49,6)
Trabalha						
Sim	32,0	(30,5–33,5)	27,1	(25,2–29,0)	37,8	(35,6–40,1)
Não	34,6	(32,3–36,9)	26,2	(23,3–29,4)	42,3	(38,9–45,7)
Situação do domicílio						
Urbano	32,6	(31,2–34,0)	26,7	(24,9–28,6)	38,7	(36,7–40,7)
Rural	33,9	(31,1–36,9)	27,6	(24,6–30,9)	45,2	(40,1–50,4)
Grandes regiões						
Norte	23,7	(21,4–26,1)	19,0	(16,2–22,1)	29,4	(26,1–32,9)
Nordeste	33,8	(31,9–35,7)	26,7	(24,5–28,9)	41,5	(38,5–44,5)
Sudeste	33,1	(30,7–35,5)	28,0	(24,9–31,3)	38,6	(35,2–42,2)
Sul	33,8	(31,3–36,4)	26,8	(23,6–30,3)	41,2	(37,3–45,2)
Centro-Oeste	34,3	(30,9–37,9)	29,0	(24,6–33,9)	40,0	(35,8–44,4)

SM: salário mínimo.

**Tabela 2:** Prevalência do não uso de preservativo na última relação sexual e respectivos intervalos de confiança de 95% das pessoas de 18 anos ou mais casadas ou em coabitação, por sexo, segundo características socioeconômicas, demográficas e regionais. Pesquisa Nacional de Saúde, Brasil, 2019.

Características socioeconômicas, demográficas e regionais	Casados ou em coabitação					
	Total		Homens (n = 23.010)		Mulheres (n = 19.231)	
Total	75,0	(74,3–75,7)	75,0	(74,0–75,9)	75,0	(74,0–76,1)
Faixa etária						
18–24 anos	56,4	(53,3–59,4)	55,7	(50,7–60,7)	56,8	(53,2–60,4)
25–29 anos	62,7	(60,1–65,2)	62,1	(58,7–65,4)	63,2	(59,5–66,7)
30–39 anos	70,6	(69,2–72,1)	68,9	(66,9–70,9)	72,3	(70,3–74,3)
40–49 anos	75,4	(74,1–76,7)	74,7	(72,7–76,7)	76,1	(74,2–78,0)
50–59 anos	83,8	(82,4–85,1)	82,4	(80,5–84,1)	85,6	(83,5–87,5)
≥ 60 anos	89,8	(88,5–91,0)	88,5	(86,9–89,9)	92,4	(89,8–94,4)
Cor ou raça						
Branca	77,0	(75,9–78,0)	77,4	(75,9–78,7)	76,6	(75,0–78,1)
Preta	72,3	(70,1–74,4)	71,7	(68,6–74,6)	73,0	(69,8–76,0)
Parda	73,8	(72,8–74,8)	73,5	(72,2–74,8)	74,1	(72,6–75,6)
Nível de instrução						
Sem instrução ou fundamental incompleto	80,0	(78,9–81,1)	79,0	(77,6–80,4)	81,4	(79,6–83,1)
Fundamental completo ou médio incompleto	73,3	(71,4–75,2)	73,1	(70,6–75,5)	73,6	(70,7–76,3)
Médio completo ou superior incompleto	71,3	(70,1–72,5)	71,4	(69,6–73,1)	71,3	(69,5–72,9)
Superior completo	75,0	(73,5–76,5)	75,8	(73,3–78)	74,4	(72,2–76,5)
Rendimento domiciliar per capita						
Até 1 SM	73,2	(72,2–74,2)	72,6	(71,2–73,9)	73,8	(72,4–75,2)
Mais de 1 a 3 SM	76,5	(75,4–77,6)	76,8	(75,3–78,3)	76,1	(74,3–77,8)
Mais de 3 a 5 SM	76,8	(74,0–79,5)	78,3	(74,3–81,7)	75,0	(70,6–79,0)
Mais de 5 SM	79,5	(77,0–81,8)	78,8	(75,4–81,9)	80,3	(76,3–83,8)
Trabalha						
Sim	73,7	(72,8–74,5)	73,4	(72,3–74,5)	74,1	(72,8–75,4)
Não	78,1	(76,9–79,3)	82,0	(80,0–83,8)	76,3	(74,7–77,8)
Situação do domicílio						
Urbano	74,4	(73,6–75,2)	74,7	(73,6–75,8)	74,2	(72,9–75,3)
Rural	78,1	(76,8–79,3)	76,3	(74,6–78,0)	80,3	(78,6–81,8)
Grandes regiões						
Norte	66,1	(64,1–68,0)	65,0	(62,2–67,6)	67,3	(64,9–69,6)
Nordeste	73,3	(72,1–74,4)	72,5	(70,9–74,0)	74,1	(72,6–75,6)
Sudeste	76,5	(75,2–77,8)	76,6	(74,8–78,3)	76,4	(74,3–78,3)
Sul	77,1	(75,5–78,6)	77,8	(75,8–79,7)	76,2	(73,9–78,4)
Centro-Oeste	77,7	(76,1–79,3)	79,1	(76,9–81,2)	76,2	(73,9–78,4)

SM: salário mínimo.

Do total de entrevistados, 69.331 pessoas informaram a idade de sua iniciação sexual, enquanto 13.021 (14,7%) não souberam dar essa informação ou não lembravam, 1.624 (1,8%) nunca tinham tido relação sexual na vida e 4.555 (5,1%) se recusaram a responder à pergunta. Na faixa de 18 a 24 anos de idade, o total de entrevistados com informação da iniciação sexual foi de 5.955 pessoas de ambos os sexos (Tabela 3).

**Tabela 3:** Prevalência de iniciação sexual precoce e respectivos intervalos de confiança de 95% das pessoas de 18 a 24 anos de idade, por sexo, segundo características socioeconômicas, demográficas e regionais. Pesquisa Nacional de Saúde, Brasil, 2019.

Características socioeconômicas e demográficas	Total	Homens (n = 2.764)	Mulheres (n = 3.191)
Total	24,2 (22,4–26,1)	28,5 (25,6–31,6)	19,9 (18,0–22,1)
Cor ou raça			
Branca	19,5 (16,6–22,7)	23,5 (19,0–28,6)	15,7 (12,6–19,4)
Preta	25,7 (21,1–30,9)	32,7 (25,2–41,1)	19,6 (14,1–26,7)
Parda	27,5 (25,0–30,2)	31,6 (27,5–36,0)	23,3 (20,4–26,4)
Outras	26,9 (13,2–47,1)	22,0 (6,4–53,8)	31,8 (16,3–52,7)
Nível de instrução			
Sem instrução ou fundamental incompleto	46,7 (41,1–52,4)	46,4 (38,3–54,7)	47,0 (40,0–54,2)
Fundamental completo ou médio incompleto	32,3 (28,3–36,5)	36,6 (31–42,5)	27,2 (22,4–32,6)
Médio completo ou acima	17,2 (15,1–19,5)	21,0 (17,6–24,9)	13,9 (11,7–16,5)
Rendimento domiciliar per capita			
Até 1 SM	27,9 (25,5–30,4)	32,9 (28,9–37,2)	23,3 (20,7–26,1)
Mais de 1 a 3 SM	20,2 (17,3–23,4)	24,3 (20–29,2)	15,9 (12,3–20,3)
Mais de 3 a 5 SM	11,5 (5,8–21,4)	15,4 (6,8–31,2)	6,1 (2,2–15,8)
Mais de 5 SM	8,0 (3,6–16,9)	12,3 (5,3–26,1)	0,6 (0,1–2,6)
Situação do domicílio			
Urbano	24,2 (22,2–26,3)	28,9 (25,7–32,4)	19,6 (17,4–22,0)
Rural	24,1 (20,7–27,8)	26,0 (21,1–31,5)	22,0 (17,6–27,1)
Grandes regiões			
Norte	25,8 (22,6–29,3)	31,4 (26,2–37,2)	20,4 (16,7–24,8)
Nordeste	28,1 (25,2–31,3)	32,7 (27,9–37,8)	23,6 (20,0–27,8)
Sudeste	21,7 (18,4–25,5)	28,0 (22,6–34,1)	15,6 (12,3–19,4)
Sul	20,9 (17,3–25,0)	21,1 (15,9–27,6)	20,7 (15,7–26,7)
Centro-Oeste	27,5 (22,7–33,0)	26,6 (20,2–34,1)	28,4 (21,7–36,3)

SM: salário mínimo.

Com relação ao não uso de preservativo, foram calculadas as prevalências separadamente para pessoas não coabitantes e aquelas casadas ou em coabitação e por sexo (Tabelas 1 e 2). O não uso de preservativo foi consideravelmente maior entre as pessoas casadas ou em coabitação, chegando a 75% (IC95% 74,3–75,7) em ambos os sexos. Já entre as pessoas não coabitantes, esse percentual foi de 26,9% (IC95% 25,2–28,5) e 39,1% (IC95% 37,3–41,0), para homens e mulheres, respectivamente.

Independente do estado civil, o não uso de preservativo foi significativamente maior entre as faixas etárias mais elevadas e pessoas sem instrução ou com nível fundamental incompleto. Vale ainda ressaltar que, entre as mulheres casadas ou que coabitam, com idade igual ou superior a 60 anos, foi observada a maior prevalência do não uso de preservativo (92,4%; IC95% 89,8–94,4) dentre todas as categorias analisadas. Além disso, o não uso de preservativo foi significativamente maior entre as mulheres casadas/coabitantes da região rural (80,3%; IC95% 78,6–81,8), quando comparadas às da região urbana (74,2%; IC95% 72,9–75,3).

Com relação ainda ao não uso de preservativo, ao comparar as categorias de cada uma das demais variáveis socioeconômicas, demográficas e regionais não mencionadas anteriormente, foram encontradas diferenças estatisticamente significativas também nos seguintes casos: i) homens casados/coabitantes, brancos, com maior percentual de não uso de preservativo , quando comparados aos pretos e pardos; ii) homens não coabitantes e homens e mulheres casados/coabitantes com RDPC maior que cinco SM, apresentando maior prevalência que aqueles com RDPC de até um SM; iii) homens casados/coabitantes, que não trabalham, com prevalência maior que os que trabalham; iv) pessoas da região norte apresentando não uso de preservativo menor que as pessoas das demais regiões para ambos os sexos e estado civil.

As diferenças do não uso do preservativo entre homens e mulheres, para as diversas características analisadas, se mostraram relevantes no grupo dos não coabitantes. As prevalências do não uso de preservativo foram significativamente diferentes entre os sexos em todas as categorias analisadas, com exceção daquelas referentes às pessoas com idade entre 25 a 29 anos e dos que residem em domicílios cujo RDPC era acima de três SM (Tabela 1).

Destaca-se, por exemplo, a disparidade observada entre pessoas com idade entre 30 e 39 anos, faixa em que a prevalência do não uso de preservativo entre os homens foi de 22,6% (IC95% 19,6–26,0), enquanto entre as mulheres chegou a 39,6% (IC95% 36,5–42,7). Entre pessoas com nível fundamental completo ou médio incompleto os valores chegam a 21,8% (IC95% 18,3–25,7) e 39,4% (IC95% 34,7–44,2), respectivamente.

Com relação à iniciação sexual, verificou-se que homens iniciam a vida sexual mais precocemente que mulheres (Figura 2). No Brasil, aproximadamente 24% dos homens de 18 anos ou mais de idade iniciaram a vida sexual precocemente, enquanto entre as mulheres essa prevalência chegou a 10,8%.

**Figura 2:** Prevalência de iniciação sexual precoce e intervalos de confiança de 95% das pessoas de 18 anos de idade ou mais, por sexo, segundo faixas etárias. Pesquisa Nacional de Saúde, Brasil, 2019.

Conforme pode ser observado na Figura 2, a disparidade entre os sexos vem diminuindo a cada geração. A diferença das prevalências de iniciação sexual precoce entre homens e mulheres foram de 16,5% e 14,4%, respectivamente, para pessoas de 50 a 59 anos e 60 anos ou mais de idade. No entanto, ela diminui ao longo das faixas etárias mais jovens chegando a 7,1% dentre as pessoas de 25 a 29 anos, e 8,6% entre aqueles de 18 a 24 anos de idade.

Considerando a população de 18 a 24 anos de idade, os dados revelam ainda que para as mulheres a iniciação sexual precoce é significativamente maior também entre as pessoas pardas, quando comparadas às brancas. Entre as de cor parda, a prevalência chega a mais de sete pontos percentuais acima da observada entre as de cor branca (Tabela 3).

Observou-se também que, para ambos os sexos, as menores taxas de iniciação sexual precoce estavam entre as pessoas com maiores graus instrucionais. Enquanto a prevalência foi próxima de 47% entre aqueles sem instrução ou com nível fundamental incompleto para ambos os sexos, entre as pessoas com nível médio completo ou acima, ela foi de 21,0% e 13,9% para homens e mulheres, respectivamente.

Resultados similares são obtidos ao analisar a iniciação sexual precoce segundo faixas de RDPC, em que a menor prevalência observada está no grupo das mulheres que residem em domicílios com renda per capita acima de 5 SM (0,6%; IC95% 0,1–2,6). Nesse grupo a prevalência de iniciação sexual precoce entre homens foi de 12,3% (IC95% 5,3–26,1).

Ao avaliar os resultados obtidos separados por macrorregiões do país é possível destacar a região Sudeste, onde a prevalência de iniciação sexual precoce entre os homens foi de 28% (IC95% 22,6–34,1) enquanto entre as mulheres chegou a 15,6% (IC95% 12,3–19,4).

## DISCUSSÃO

Após mais de cinco anos sem estatísticas oficiais de órgãos governamentais a respeito dos comportamentos sexuais de risco da população adulta em escala nacional, a inclusão do novo módulo de atividade sexual na PNS 2019 viabilizou a obtenção de um panorama mais atual sobre o tema, permitindo inclusive a obtenção de resultados para as regiões urbana e rural, bem como para idosos.

A última pesquisa realizada sobre o tema que se tem conhecimento foi a Pesquisa de Conhecimentos, Atitudes e Práticas da População Brasileira, realizada pelo Ministério da Saúde em 2013 (PCAP - 2013), com foco na população com idades entre 15 e 64 anos.

Os resultados obtidos no presente estudo, indicam que o não uso de preservativo na última relação sexual foi significativamente maior entre pessoas casadas ou que coabitam com o parceiro (75%) e entre os mais velhos, chegando, por exemplo, a 92,4% entre mulheres casadas/coabitantes com 60 anos de idade ou mais, sendo a maior prevalência de todas as faixas etárias e demais características avaliadas.

A inclusão dos idosos na pesquisa mostra-se de grande relevância, uma vez que se tem observado nos últimos anos um aumento na taxa de detecção de aids entre homens com 60 anos ou mais, segundo o último boletim epidemiológico sobre o tema^2^.

Os resultados apresentados no relatório da PCAP - 2013, apesar de não haver a desagregação por sexo e estado civil simultaneamente, e se restringir à população com idades entre 15 e 64 anos, corroboram o fato de o não uso de preservativo ser maior entre pessoas casadas/coabitantes, com níveis de instrução menores e de faixas etárias mais avançadas^10^.

No presente estudo, para o grupo dos não coabitantes, observou-se disparidades significativas entre os sexos. Os achados estão em concordância com outros estudos da população adulta, no Brasil e em outros países da América do Norte, que apontam para um menor uso de preservativo entre as mulheres^7,9,10,19,20^. No entanto, são raros os que analisaram os adultos estratificando-os simultaneamente por sexo e estado civil.

O único estudo encontrado na literatura trata-se de um inquérito nacional de pessoas entre 18 e 44 anos nos Estados Unidos, em que o não uso de preservativo entre mulheres e homens não coabitantes foi de, respectivamente, 63,2% (IC95% 61,1–65,2) e 49% (IC95% 46,8–51,1). Apesar dos intervalos de confiança não estarem sobrepostos entre homens e mulheres casados/coabitantes, as prevalências se mostraram muito próximas, chegando a 84,3% no caso dos homens e 86,9% para as mulheres^21^.

No que se refere à iniciação sexual precoce, o presente estudo traz uma colaboração importante, por avaliar esse desfecho nas distintas gerações da população de 18 anos ou mais para ambos os sexos.

Ao comparar as prevalências de iniciação sexual precoce entre homens e mulheres, foi possível notar uma diminuição expressiva entre os sexos para as gerações mais novas quando comparadas às gerações mais velhas. O declínio na idade da menarca ao longo das gerações, bem como mudanças nas normas sexuais, pode estar entre as possíveis explicações para esse achado^22,23^.

Os resultados da PCAP - 2013, a qual utilizou o mesmo critério de iniciação sexual precoce antes dos 15 anos, também indicam uma iniciação sexual mais precoce para as gerações mais novas. No entanto, as faixas etárias analisadas foram mais restritas e não houve análise estratificada por sexo^10^. Outros estudos de base populacional focalizados nas pessoas adultas conduzidos nos EUA e Tailândia, também corroboram esse achado, além de haver indícios de um aumento de parceiros sexuais ao longo da vida para as mulheres de gerações mais novas^23,24^.

Observou-se ainda o papel do nível instrucional e do rendimento domiciliar per capita na iniciação sexual precoce. Foram observadas maiores prevalências de iniciação sexual precoce dentre os indivíduos com níveis de instrução mais baixos e residentes em domicílios com menores RDPC. Tal resultado reforça a importância da educação de uma maneira geral e da educação sexual nas escolas para a prevenção de IST entre os mais jovens, como já apontado em outros estudos^17,24,25^.

Como potencialidade deste estudo, considera-se o fato dos dados utilizados serem oriundos de um inquérito de base populacional em escala nacional, com foco na população adulta, menos explorada na temática de comportamentos sexuais de risco. Além disso, o desfecho do uso do preservativo na última relação sexual permite uma melhor comparação com os resultados de outros países, pois é uma medida amplamente utilizada em inquéritos sobre o tema, além de uma boa *proxy* do uso de preservativo ao longo do tempo^4,21^.

Foi possível obter ainda resultados para a população idosa, diferentemente de grande parte das demais pesquisas nessa temática, isso porque de maneira geral os estudos estabelecem um limite de idade máximo durante a coleta de dados. Por fim, a análise do uso de preservativo estratificada simultaneamente por sexo e estado civil é inédita. Não foram identificados estudos no Brasil para a população adulta em escala nacional com esse delineamento.

Entre as limitações, está um possível viés de memória mais intenso para as pessoas de maior faixa etária, especialmente quanto à lembrança da informação da idade de iniciação sexual. Ademais, as características socioeconômicas da população, com idades entre 18 e 24 anos, informadas no momento da coleta foram consideradas *proxy* daquelas referentes ao momento da iniciação sexual.

O objetivo do presente estudo foi descrever os comportamentos sexuais de risco dos adultos brasileiros, segundo características socioeconômicas, demográficas e regionais. Os resultados obtidos evidenciam disparidades relevantes entre os sexos. Destaca-se ainda a alta prevalência do não uso de preservativo na população mais velha, também exposta às IST e que precisa ser considerada nos esforços da promoção de saúde. Por fim, o aumento da prevalência da iniciação sexual precoce entre as mulheres das gerações mais novas mostra-se preocupante do ponto de vista de saúde pública, podendo implicar em um aumento de gravidezes indesejadas e de IST.

Os resultados obtidos são de extrema importância para dar visibilidade aos estratos da população adulta atualmente mais vulneráveis às IST e fundamentar estudos futuros sobre o tema, além de apontarem a necessidade de políticas públicas voltadas para a redução das disparidades entre os sexos relacionadas aos comportamentos sexuais de risco.
