# Supplementary material for: Erratum
Source: Rev Saude Publica. 2022 Nov 18;56:111. doi: 10.11606/S01518-8787.2022056004007err (PMC9749661; doi:10.11606/S01518-8787.2022056004007err)
Supplement: Translation

# Translation: Errata

No artigo “Panorama dos comportamentos sexuais de risco na população adulta brasileira - PNS 2019”, DOI , publicado na Revista de Saúde Pública.2022;56:61, nas páginas 1, 4, 5, 6, 7 e 9 a RSP corrige as tabelas 1 e 2, a seção resultados e estimativas informadas no resumo e na discussão.

**Onde se lê:**

**Table:** Tabela 1 (p. 5):

Características socioeconômicas, demográficas e regionais	Não coabitantes					

	Total		Homens (n = 10.076)		Mulheres (n = 9.006)	
Total	32,7	(31,5–34,0)	26,9	(25,2–28,5)	39,1	(37,3–41,0)
Faixa etária						
18–24 anos	19,9	(17,5–22,6)	15,2	(12,5–18,3)	26,2	(22,0–30,9)
25–29 anos	27,5	(24,1–31,2)	23,0	(18,6–28,0)	32,7	(27,9–38,0)
30–39 anos	31,6	(29,3–33,9)	22,6	(19,6–26,0)	39,6	(36,5–42,7)
40–49 anos	40,5	(37,7–43,3)	32,8	(29,0–36,8)	45,8	(42,1–49,6)
50–59 anos	50,0	(46,7–53,3)	45,4	(40,7–50,1)	55,2	(50,8–59,5)
≥ 60 anos	59,7	(56,1–63,3)	55,7	(51,4–60,0)	67,6	(61,1–73,4)
Cor ou raça						
Branca	34,6	(32,7–36,6)	28,5	(26,0–31,1)	40,9	(37,9–43,9)
Preta	31,3	(28,0–34,8)	23,7	(19,4–28,6)	39,4	(34,2–44,7)
Parda	31,5	(29,8–33,4)	26,6	(24,2–29,1)	37,4	(34,9–40,0)
Nível de instrução						
Sem instrução ou fundamental incompleto	43,0	(40,6–45,4)	36,6	(33,5–39,7)	52,2	(48,4–56,0)
Fundamental completo ou médio incompleto	28,9	(25,9–32,1)	21,8	(18,3–25,7)	39,4	(34,7–44,2)
Médio completo ou superior incompleto	28,8	(26,9–30,9)	23,8	(21,2–26,5)	34,0	(31,1–37,1)
Superior completo	34,3	(31,6–37,1)	27,2	(23,5–31,3)	39,5	(35,9–43,2)
Rendimento domiciliar per capita						
Até 1 SM	32,0	(30,2–33,8)	24,5	(22,0–27,1)	38,6	(36,1–41,1)
Mais de 1 a 3 SM	32,7	(30,7–34,8)	27,6	(25,0–30,3)	39,7	(36,4–43,1)
Mais de 3 a 5 SM	33,2	(28,9–37,9)	29,9	(24,8–35,6)	37,6	(30,5–45,2)
Mais de 5 SM	37,4	(33,4–41,5)	33,5	(28,4–38,9)	43,0	(36,7–49,6)
Trabalha						
Sim	32,0	(30,5–33,5)	27,1	(25,2–29,0)	37,8	(35,6–40,1)
Não	34,6	(32,3–36,9)	26,2	(23,3–29,4)	42,3	(38,9–45,7)
Situação do domicílio						
Urbano	32,6	(31,2–34,0)	26,7	(24,9–28,6)	38,7	(36,7–40,7)
Rural	33,9	(31,1–36,9)	27,6	(24,6–30,9)	45,2	(40,1–50,4)
Grandes regiões						
Norte	23,7	(21,4–26,1)	19,0	(16,2–22,1)	29,4	(26,1–32,9)
Nordeste	33,8	(31,9–35,7)	26,7	(24,5–28,9)	41,5	(38,5–44,5)
Sudeste	33,1	(30,7–35,5)	28,0	(24,9–31,3)	38,6	(35,2–42,2)
Sul	33,8	(31,3–36,4)	26,8	(23,6–30,3)	41,2	(37,3–45,2)
Centro–Oeste	34,3	(30,9–37,9)	29,0	(24,6–33,9)	40,0	(35,8–44,4)

**Leia-se:**

**Table:** Tabela 1 (p. 5):

Características socioeconômicas, demográficas e regionais	Não coabitantes					

	Total (n = 17.376)		Homens (n = 9.162)		Mulheres (n = 8.214)	
Total	27,3	(26,0–28,7)	21,3	(19,6–23,0)	34,1	(32,2–36,1)
Faixa etária						
18–24 anos	19,8	(17,4–22,6)	15,2	(12,5–18,3)	26,1	(21,9–30,7)
25–29 anos	26,6	(23,2–30,4)	22,4	(18,0–27,4)	31,8	(26,8–37,2)
30–39 anos	27,6	(25,3–29,9)	18,9	(16,0–22,1)	35,5	(32,3–38,8)
40–49 anos	32,4	(29,8–35,1)	23,4	(20,1–27,1)	38,7	(35,0–42,6)
50–59 anos	39,9	(36,5–43,3)	34,1	(29,8–38,8)	46,0	(41,2–51,0)
≥ 60 anos	43,8	(39,8–47,9)	39,9	(35,4–44,5)	52,0	(44,2–59,8)
Cor ou raça						
Branca	28,9	(26,8–31,0)	22,7	(20,2–25,4)	35,3	(32,2–38,5)
Preta	28,0	(24,7–31,5)	20,1	(15,9–25,1)	36,1	(30,9–41,5)
Parda	25,9	(24,1–27,8)	20,6	(18,4–23,1)	32,2	(29,7–34,8)
Nível de instrução						
Sem instrução ou fundamental incompleto	33,7	(31,2–36,3)	26,8	(23,8–30,0)	43,8	(39,8–48,0)
Fundamental completo ou médio incompleto	24,2	(21,2–27,4)	17,9	(14,5–21,8)	34,0	(29,6–38,8)
Médio completo ou superior incompleto	24,9	(22,9–27,0)	20,0	(17,4–22,9)	30,0	(27,0–33,1)
Superior completo	29,9	(27,2–32,8)	21,4	(18,0–25,2)	36,2	(32,4–40,1)
Rendimento domiciliar per capita						
Até 1 SM	27,5	(25,7–29,4)	18,9	(16,4–21,6)	34,9	(32,4–37,6)
Mais de 1 a 3 SM	26,5	(24,4–28,7)	22,2	(19,6–25,0)	32,7	(29,3–36,3)
Mais de 3 a 5 SM	27,8	(23,4–32,6)	24,3	(19,2–30,2)	32,5	(25,3–40,6)
Mais de 5 SM	30,2	(26,4–34,3)	26,7	(22,0–32,1)	35,4	(28,9–42,6)
Trabalha						
Sim	26,9	(25,4–28,6)	21,6	(19,8–23,6)	33,2	(30,8–35,6)
Não	28,4	(26,2–30,8)	20,2	(17,4–23,3)	36,2	(32,8–39,8)
Situação do domicílio						
Urbano	27,4	(26,0–28,8)	21,3	(19,5–23,2)	33,8	(31,7–35,8)
Rural	27,1	(24,3–30,2)	21,2	(18,3–24,5)	38,4	(33,0–44,0)
Grandes regiões						
Norte	20,5	(18,2–23,0)	16,1	(13,4–19,2)	25,9	(22,6–29,5)
Nordeste	29,0	(27,1–31,1)	21,0	(18,8–23,3)	37,7	(34,7–40,9)
Sudeste	28,1	(25,7–30,7)	22,9	(19,8–26,3)	33,9	(30,4–37,7)
Sul	25,3	(22,8–28,0)	19,2	(16,0–22,8)	32,2	(28,3–36,4)
Centro–Oeste	27,7	(24,3–31,4)	22,1	(17,9–27,0)	33,9	(29,7–38,2)

**Onde se lê:**

**Table:** Tabela 2 (p. 6):

Características socioeconômicas, demográficas e regionais	Casados ou em coabitação					

	Total		Homens (n = 23.010)		Mulheres (n = 19.231)	
Total	75,0	(74,3–75,7)	75,0	(74,0–75,9)	75,0	(74,0–76,1)
Faixa etária						
18–24 anos	56,4	(53,3–59,4)	55,7	(50,7–60,7)	56,8	(53,2–60,4)
25–29 anos	62,7	(60,1–65,2)	62,1	(58,7–65,4)	63,2	(59,5–66,7)
30–39 anos	70,6	(69,2–72,1)	68,9	(66,9–70,9)	72,3	(70,3–74,3)
40–49 anos	75,4	(74,1–76,7)	74,7	(72,7–76,7)	76,1	(74,2–78,0)
50–59 anos	83,8	(82,4–85,1)	82,4	(80,5–84,1)	85,6	(83,5–87,5)
≥ 60 anos	89,8	(88,5–91,0)	88,5	(86,9–89,9)	92,4	(89,8–94,4)
Cor ou raça						
Branca	77,0	(75,9–78,0)	77,4	(75,9–78,7)	76,6	(75,0–78,1)
Preta	72,3	(70,1–74,4)	71,7	(68,6–74,6)	73,0	(69,8–76,0)
Parda	73,8	(72,8–74,8)	73,5	(72,2–74,8)	74,1	(72,6–75,6)
Nível de instrução						
Sem instrução ou fundamental incompleto	80,0	(78,9–81,1)	79,0	(77,6–80,4)	81,4	(79,6–83,1)
Fundamental completo ou médio incompleto	73,3	(71,4–75,2)	73,1	(70,6–75,5)	73,6	(70,7–76,3)
Médio completo ou superior incompleto	71,3	(70,1–72,5)	71,4	(69,6–73,1)	71,3	(69,5–72,9)
Superior completo	75,0	(73,5–76,5)	75,8	(73,3–78)	74,4	(72,2–76,5)
Rendimento domiciliar per capita						
Até 1 SM	73,2	(72,2–74,2)	72,6	(71,2–73,9)	73,8	(72,4–75,2)
Mais de 1 a 3 SM	76,5	(75,4–77,6)	76,8	(75,3–78,3)	76,1	(74,3–77,8)
Mais de 3 a 5 SM	76,8	(74,0–79,5)	78,3	(74,3–81,7)	75,0	(70,6–79,0)
Mais de 5 SM	79,5	(77,0–81,8)	78,8	(75,4–81,9)	80,3	(76,3–83,8)
Trabalha						
Sim	73,7	(72,8–74,5)	73,4	(72,3–74,5)	74,1	(72,8–75,4)
Não	78,1	(76,9–79,3)	82,0	(80,0–83,8)	76,3	(74,7–77,8)
Situação do domicílio						
Urbano	74,4	(73,6–75,2)	74,7	(73,6–75,8)	74,2	(72,9–75,3)
Rural	78,1	(76,8–79,3)	76,3	(74,6–78,0)	80,3	(78,6–81,8)
Grandes regiões						
Norte	66,1	(64,1–68,0)	65,0	(62,2–67,6)	67,3	(64,9–69,6)
Nordeste	73,3	(72,1–74,4)	72,5	(70,9–74,0)	74,1	(72,6–75,6)
Sudeste	76,5	(75,2–77,8)	76,6	(74,8–78,3)	76,4	(74,3–78,3)
Sul	77,1	(75,5–78,6)	77,8	(75,8–79,7)	76,2	(73,9–78,4)
Centro–Oeste	77,7	(76,1–79,3)	79,1	(76,9–81,2)	76,2	(73,9–78,4)

**Leia-se:**

**Table:** Tabela 2 (p. 6):

Características socioeconômicas, demográficas e regionais	Casados ou em coabitação					

	Total (n = 43.947)		Homens (n = 23.924)		Mulheres (n = 20.023)	
Total	75,2	(74,5–75,9)	75,1	(74,2–76,1)	75,3	(74,2–76,3)
Faixa etária						
18–24 anos	56,4	(53,3–59,4)	55,7	(50,7–60,6)	56,8	(53,1–60,4)
25–29 anos	62,7	(60,2–65,2)	62,3	(58,9–65,5)	63,1	(59,5–66,6)
30–39 anos	70,7	(69,2–72,1)	68,9	(66,8–70,8)	72,5	(70,5–74,4)
40–49 anos	75,5	(74,2–76,8)	74,8	(72,8–76,7)	76,1	(74,2–78,0)
50–59 anos	83,7	(82,0–85,0)	82,2	(80,1–84,1)	85,7	(83,7–87,5)
≥ 60 anos	89,8	(88,6–90,9)	88,4	(86,9–89,8)	92,5	(90,1–94,4)
Cor ou raça						
Branca	77,2	(76,2–78,2)	77,5	(76,1–78,8)	76,9	(75,3–78,4)
Preta	72,2	(69,8–74,4)	71,2	(67,9–74,3)	73,3	(70,2–76,2)
Parda	74,1	(73,1–75,1)	73,9	(72,6–75,2)	74,3	(72,8–75,7)
Nível de instrução						
Sem instrução ou fundamental incompleto	80,3	(79,2–81,4)	79,3	(77,9–80,6)	81,7	(79,9–83,3)
Fundamental completo ou médio incompleto	73,1	(71,2–74,9)	72,6	(70,1–75,0)	73,6	(70,8–76,3)
Médio completo ou superior incompleto	71,6	(70,4–72,8)	71,5	(69,8–73,2)	71,7	(70,0–73,3)
Superior completo	75,2	(73,7–76,7)	76,2	(73,8–78,4)	74,4	(72,2–76,5)
Rendimento domiciliar per capita						
Até 1 SM	73,4	(72,4–74,4)	72,9	(71,5–74,1)	74,0	(72,6–75,3)
Mais de 1 a 3 SM	76,6	(75,4–77,7)	76,8	(75,1–78,4)	76,4	(74,6–78,1)
Mais de 3 a 5 SM	77,0	(74,2–79,6)	78,5	(74,7–81,9)	75,0	(70,8–78,9)
Mais de 5 SM	80,1	(77,7–82,3)	79,4	(76,0–82,4)	81,0	(77,2–84,2)
Trabalha						
Sim	73,9	(73,0–74,7)	73,5	(72,4–74,6)	74,3	(73,0–75,6)
Não	78,5	(77,2–79,6)	82,3	(80,4–84,0)	76,6	(75,1–78,1)
Situação do domicílio						
Urbano	74,6	(73,9–75,4)	74,8	(73,7–75,9)	74,4	(73,3–75,6)
Rural	78,4	(77,1–79,6)	76,7	(74,9–78,4)	80,4	(78,8–82,0)
Grandes regiões						
Norte	66,3	(64,4–68,2)	65,2	(62,5–67,8)	67,6	(65,3–69,9)
Nordeste	73,6	(72,4–74,7)	72,8	(71,3–74,3)	74,4	(72,9–75,9)
Sudeste	76,5	(75,3–77,7)	76,6	(74,7–78,3)	76,5	(74,5–78,4)
Sul	77,3	(75,7–78,8)	78,0	(76,0–79,9)	76,6	(74,3–78,7)
Centro–Oeste	78,2	(76,6–79,7)	79,5	(77,4–81,5)	76,7	(74,4–78,7)

**Onde se lê:**

Resumo (p. 1):

“A prevalência do não uso de preservativo entre casados/coabitantes foi igual em ambos os sexos (75,0%).”

“39,1%”; “26,9%”

**Leia-se:**

Resumo (p. 1):

“A prevalência do não uso de preservativo entre casados/coabitantes foi similar em ambos os sexos (75,1% e 75,3%, entre homens e mulheres).”

“34,1%”; “21,3%.”

**Onde se lê:**

Seção Resultados:

(último parágrafo, p. 4)

“75% (IC95% 74,3–75,7)”; “26,9% (IC95% 25,2–28,5)”; “39,1% (IC95% 37,3–41,0)”

**Leia-se:**

Seção Resultados:

(último parágrafo, p. 4)

“75,2% (IC95% 74,5–75,9)”; “21,3% (IC95% 19,6–23,0)”; “34,1% (IC95% 32,2–36,1)”

**Onde se lê:**

(último parágrafo, p. 5)

“Independente do estado civil, o não uso de preservativo foi significativamente maior entre as faixas etárias mais elevadas e pessoas sem instrução ou com nível fundamental incompleto.”

“(92,4%; IC95% 89,9–94,4)”; “(80,3%; IC95% 78,6–81,8)”; (74,2%; IC95% 72,9–75,3).”

**Leia-se:**

(último parágrafo, p. 5)

“Independente do estado civil, o não uso de preservativo foi significativamente maior entre as faixas etárias mais elevadas.”

“(92,5%; IC95% 90,1–94,4)”; “(80,4%; IC95% 78,8–82,0)”; (74,4%; IC95% 73,3–75,6).”

**Onde se lê:**

(1º parágrafo, p. 7)

“iv) pessoas da região norte apresentando não uso de preservativo menor que as pessoas das demais regiões para ambos os sexos e estado civil.”

**Leia-se:**

(1º parágrafo, p. 7)

“iv) pessoas da região norte casadas ou em coabitação apresentando não uso de preservativo menor que as pessoas das demais regiões para ambos os sexos.”

**Onde se lê:**

(2º parágrafo, p. 7)

“com exceção daquelas referentes às pessoas com idade entre 25 a 29 anos e dos que residem em domicílios cujo RDPC era acima de três SM (Tabela 1)”.

**Leia-se:**

(2º parágrafo, p. 7)

“com exceção daquelas referentes às pessoas com idade entre 25 a 29 anos, maior que 60 anos e dos que residem em domicílios cujo RDPC era acima de três SM (Tabela 1).”

**Onde se lê:**

(3º parágrafo, p. 7)

“Destaca-se, por exemplo, a disparidade observada entre pessoas com idade entre 30 e 39 anos, faixa em que a prevalência do não uso de preservativo entre os homens foi de 22,6% (IC95% 19,6–26,0), enquanto entre as mulheres chegou a 39,6% (IC95% 36,5–42,7). Entre pessoas com nível fundamental completo ou médio incompleto os valores chegam a 21,8% (IC95% 18,3–25,7) e 39,4% (IC95% 34,7–44,2), respectivamente.”

**Leia-se:**

(3º parágrafo, p. 7)

“Destaca-se, por exemplo, a disparidade observada entre pessoas da área rural, em que a prevalência do não uso de preservativo entre os homens foi de 21,2% (IC95% 18,3–24,5), enquanto entre as mulheres chegou a 38,4% (IC95% 33,0–44,0). Entre pessoas sem instrução ou com nível fundamental incompleto os valores chegam a 26,8% (IC95% 23,8–30,0) e 43,8% (IC95% 39,8–48,0), respectivamente.”

**Onde se lê:**

Seção Discussão:

(2º parágrafo, p. 9)

“(75,0%)”; “(92,4%)”

**Leia-se:**

Seção Discussão:

(2º parágrafo, p. 9)

“(75,2%)”; “92,5%”
